# Smoking shifts human small airway epithelium club cells toward a lesser differentiated population

**DOI:** 10.1038/s41525-021-00237-1

**Published:** 2021-09-08

**Authors:** Mahboubeh R. Rostami, Michelle G. LeBlanc, Yael Strulovici-Barel, Wulin Zuo, Jason G. Mezey, Sarah L. O’Beirne, Robert J. Kaner, Philip L. Leopold, Ronald G. Crystal

**Affiliations:** 1 Department of Genetic Medicine, Weill Cornell Medical College, New York, NY USA; 2 Department of Biological Statistics and Computational Biology, Cornell University, Ithaca, NY USA; 3 Department of Medicine, Weill Cornell Medical College, New York, NY USA

**Keywords:** Molecular medicine, Biomarkers

## Abstract

The club cell, a small airway epithelial (SAE) cell, plays a central role in human lung host defense. We hypothesized that subpopulations of club cells with distinct functions may exist. The SAE of healthy nonsmokers and healthy cigarette smokers were evaluated by single-cell RNA sequencing, and unsupervised clustering revealed subpopulations of SCGCB1A1^+^KRT5^lo^MUC5AC^−^ club cells. Club cell heterogeneity was supported by evaluations of SAE tissue sections, brushed SAE cells, and in vitro air–liquid interface cultures. Three subpopulations included: (1) progenitor; (2) proliferating; and (3) effector club cells. The progenitor club cell population expressed high levels of mitochondrial, ribosomal proteins, and KRT5 relative to other club cell populations and included a differentiation branch point leading to mucous cell production. The small proliferating population expressed high levels of cyclins and proliferation markers. The effector club cell cluster expressed genes related to host defense, xenobiotic metabolism, and barrier functions associated with club cell function. Comparison of smokers vs. nonsmokers demonstrated that smoking limited the extent of differentiation of all three subclusters and altered SAM pointed domain-containing Ets transcription factor (SPDEF)-regulated transcription in the effector cell population leading to a change in the location of the branch point for mucous cell production, a potential explanation for the concomitant reduction in effector club cells and increase in mucous cells in smokers. These observations provide insights into both the makeup of human SAE club cell subpopulations and the smoking-induced changes in club cell biology.

## Introduction

The small airway epithelium (SAE) plays a central role in the early events in the pathogenesis of smoking-related lung disorders and is the first site of smoking-induced alterations in the lung^1–6^. The human SAE is composed of multiple cell types, including basal stem/progenitor cells, ciliated cells, club cells, and mucous-producing goblet cells^7,8^. Club cells comprise ~20% of the normal human SAE^9^ and express high levels of SCGB1A1 (uteroglobin, CC10)^10,11^. In contrast to the mouse, where club cells are the stem/progenitor cells, basal cells in the human SAE are the stem/progenitors, with club cells derive from the basal cell population^12–18^. Assessment of the club cell transcriptome suggests that club cells play a key role in host defense through the expression of genes involved in immune, anti-protease, anti-bacterial and physical barrier function^9,11,13,17,19–22^.

The advent of single cell transcriptomic analysis has enabled investigators to query populations of cells derived from tissues to identify subclusters of cell populations that were previously thought to be homogenous. For example, recent studies have identified distinct subclusters of airway basal and ciliated cells and alveolar type II cells based on transcriptomic profiling^23–29^. Subcluster analysis has revealed both differences within cell types in healthy and disease tissues suggesting that analysis of single cell data at higher resolution can give insight into both normal physiological functions as well as pathophysiological responses. A common environmental challenge in the human airway is exposure to cigarette smoke. Smoking causes changes in SAE basal cell differentiation with increased goblet cell numbers and decreased club and ciliated cells^30^. In order to determine whether club cells in the small airway constitute a homogenous or heterogenous population and to determine whether cigarette smoking caused changes to one or more club cell subtypes, SAE of normal nonsmoker and healthy smoker lungs were sampled by bronchoscopy and brushing, and single cell transcriptomics was utilized to identify and characterize heterogeneity in the SCGB1A1^+^ club cell population. Based on the knowledge that club cells have a defense function, this analysis was focused on defining the biology of club cells in the healthy SAE and then understanding how this biology may be altered by the stress of cigarette smoking. Using a previously published dataset^31^, subclustering of combined nonsmoker and smoker club cell populations led to the identification of three distinct, unique club cell subpopulations (progenitor, proliferating and effector). The progenitor population was identified as a branch point leading to further differentiation of both the effector population and mucous cells. Overall differentiation of the club cell lineage was reduced in smokers, leading to a change in the branch point for mucous cell production. Finally, the proportion of the differentiated club cells of the effector subpopulation was decreased and the proportion of mucous cells was increased with cigarette smoking, a finding that was linked to disrupted SAM pointed domain-containing Ets transcription factor (SPDEF) signaling in the effector cell population. Ex vivo and in vitro analyses were consistent with this finding. Furthermore, the number of proliferating club cells was increased in cigarette smokers. The presence of club cell heterogeneity suggests that specific pharmacologic targeting could be possible, leading to direct therapies to increase effector club cell numbers and overall club cell function in cigarette smokers.

## Results

### Human club cells represent a heterogeneous population

**Fig. 1:**
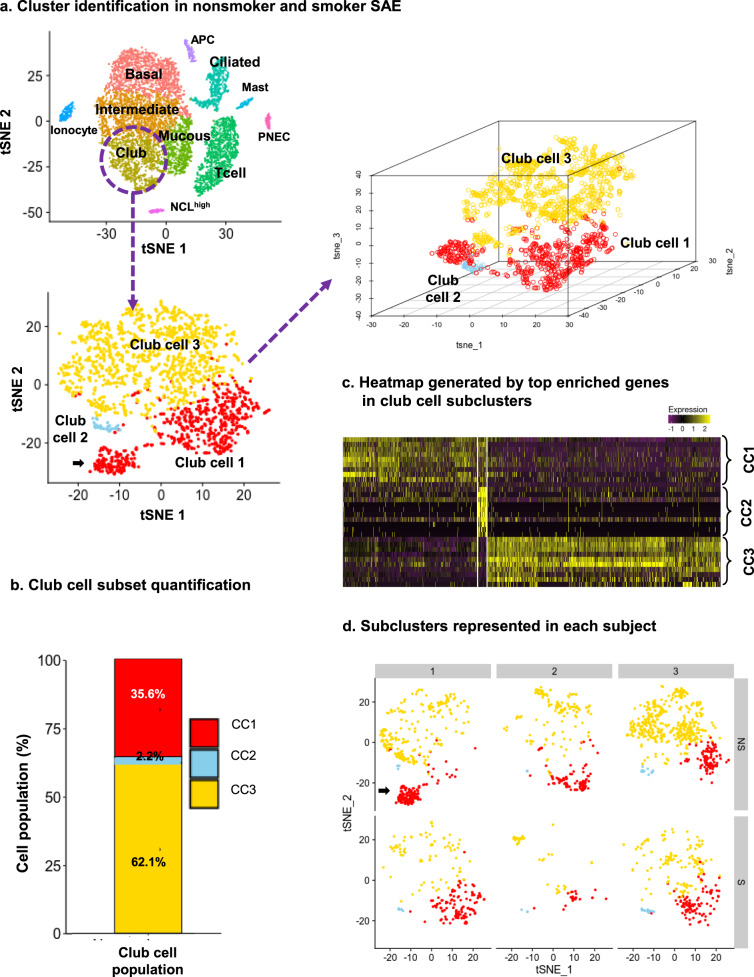
Subsets of club cells in the human small airway epithelium (SAE). **a** Cluster identification. SAE cell types were identified from Dropseq single cell RNA sequencing datasets from six individuals (three healthy nonsmokers and three healthy smokers) using established cell specific markers including KRT5 for basal cells, SCGB1A1 for club cells, MUC5AC for goblet cells, and FOXJ1/DNAI1 for ciliated cells^31^. Club cells were identified by the presence of SCGB1A1, low levels of the basal marker KRT5, and the absence of the differentiated goblet cell marker MUC5AC. Club cells underwent Seurat unsupervised clustering which identified three unique subsets of club cells: club cells 1–3 (CC1, CC2, CC3, respectively). One nonsmoker sample was enriched in pseudogenes (*arrow*); the pseudogene expression data was not used in subsequent analyses. A three-dimensional representation helps to discern the distinct localization of the three clusters in the tSNE plot. The distribution of CC1, 2 and 3 within the original low resolution cluster map of the airway cells is shown in Supplementary Fig. 1. PNEC pulmonary neuroendocrine cells, APC antigen-presenting cells; NCL^high^ represents a distinct cluster of cells identified in Zuo et al. 2020^31^. The relative size of the populations shown on the tSNE plot is not representative of the proportions of cells found in the tissue in vivo. The single-cell RNA-seq process involves separation of the epithelium into single, live cells, a process that incurs greater loss and cell death among larger cells such as ciliated cells and favors preservation of smaller cells such as basal cells. It is potentially possible for a cell type or subcluster to be under- or over-represented in this analysis. **b** Quantification of the average percent of club cells in each club cell subset relative to all club cells. **c** Heatmap of the top ten differentially expressed genes in each subcluster highlighting the differences among the three subclusters. Yellow represents increased expression levels. For the identity of genes contributing to this heatmap, see Supplementary Fig. 2. **d** Representation of each of the club cell subsets in each of the six study subjects. Note the presence of the pseudogene expression in the first nonsmoker subject (*arrow*) that was marked in panel **a** and excluded from subsequent analysis^31^.

Preliminary studies suggested that not all club cell-associated markers are distributed equally amongst all club cells in the human SAE, leading to the hypothesis that club cells are composed of multiple subtypes that could represent uniquely functioning club cell subpopulations in the SAE. To test this hypothesis, single cell sequencing was used to assess SAE samples obtained by bronchoscopy from three healthy nonsmoker and three healthy smoker subjects^31^. Using unsupervised clustering analysis, club cells were identified as cells with high expression of SCGB1A1 (expressed in both intermediate and secretory cells), low expression of KRT5 (typically expressed highly in basal cells), and the absence of MUC5AC (mucous cell-specific; Fig. 1a) i.e., SCGCB1A1+- KRT5^lo^MUC5AC^–^ cells. To evaluate club cell subpopulations, the entire club cell population underwent a separate, second round of Seurat unsupervised clustering to look for unique gene signatures in club cell subsets (Fig. 1a). In this analysis, club cells from the combined nonsmoker and smoker samples were separated into three distinct populations (club cells 1–3; Fig. 1a, b) based on the expression of specific markers (Fig. 1c, Table 1; Supplementary Fig. 2). Importantly, representatives of each of the three club cell subsets could be found in the cells obtained from each of the six study subjects (Fig. 1d).

**Table 1:** Most commonly enriched genes in each club cell subcluster.

Sub-cluster	Gene symbol	Gene name	Average log fold-change^a^	% Cells expressing in cluster	Corrected *p* value^b^
CC1	AC016739.2	AC016739.2: processed pseudogene	1.09	93	5.80 × 10^−128^
	KRT5^c^	Keratin 5	1.30	67	2.95 × 10^−123^
	MIR205HG	MIR205 host gene	1.15	84	2.89 × 10^−121^
	S100A2	S100 calcium binding protein A2	1.15	82	2.19 × 10^−117^
	CTD-3035D6.1	CTD-3035D6.1: pseudogene	0.92	78	2.39 × 10^−91^
	AC004453.8	AC004453.8: pseudogene	0.78	80	1.32 × 10^−70^
	DST	Dystonin	1.01	59	3.07 × 10^−70^
	AB019441.29	AB019441.29: processed pseudogene	0.78	59	8.05 × 10^−55^
	SERPINB13	Serpin peptidase inhibitor, member 13	0.77	52	2.12 × 10^−51^
	KRT15	Keratin 15	0.82	45	6.62 × 10^−36^
CC2	HMGB2	High mobility group box 2	2.18	90	6.15 × 10^−57^
	STMN1	Stathmin 1	2.27	93	1.56 × 10^−51^
	HIST1H4C	Histone cluster 1, H4c	1.93	90	6.07 × 10^−48^
	MKI67	Antigen identified by monoclonal antibody Ki-67	2.84	83	4.09 × 10^−47^
	TOP2A	Topoisomerase (DNA) II alpha 170 kDa	2.72	83	3.23 × 10^−44^
	CENPF	Centromere protein F; mitosin	2.67	83	1.70 × 10^−41^
	TUBA1B	Tubulin, alpha 1b	1.75	90	2.17 × 10^−37^
	NUSAP1	Nucleolar and spindle associated protein 1	1.73	58	6.62 × 10^−28^
	TPX2	TPX2, microtubule-associated	1.71	48	4.38 × 10^−22^
	CENPE	Centromere protein E, 312 kDa	1.79	40	3.73 × 10^−11^
CC3	PIGR	Polymeric immunoglobulin receptor	2.12	85	6.18 × 10^−216^
	SCGB3A1	Secretoglobin, family 3A, member 1	2.49	96	2.71 × 10^−210^
	SCGB1A1	Secretoglobin, family 1A, member 1 (uteroglobin)	1.86	99	1.37 × 10^−184^
	BPIFB1	BPI fold containing family B, member 1	2.36	82	6.65 × 10^−178^
	LCN2	Lipocalin 2	1.69	85	6.61 × 10^−151^
	C3	Complement component 3	1.49	74	6.45 × 10^−128^
	S100P	S100 calcium binding protein P	1.49	63	5.48 × 10^−108^
	MSMB	Microseminoprotein, beta-	1.78	59	3.69 × 10^−107^
	MUC5B	Mucin 5B, oligomeric mucus/gel-forming	1.75	37	9.35 × 10^−59^
	BPIFA1	BPI fold containing family A, member 1	2.92	25	1.05 × 10^−38^

^a^Average fold-change in comparison to all other groups.

^b^Bonferroni corrected *p* value.

^c^KRT5 is significantly increased in progenitor subcluster CC1, but KRT5 levels averaged across all cells in the larger combined club cell cluster are lower than KRT5 levels detected in basal cells and intermediate cells; see Supplementary Fig. 3.

**Fig. 2:**
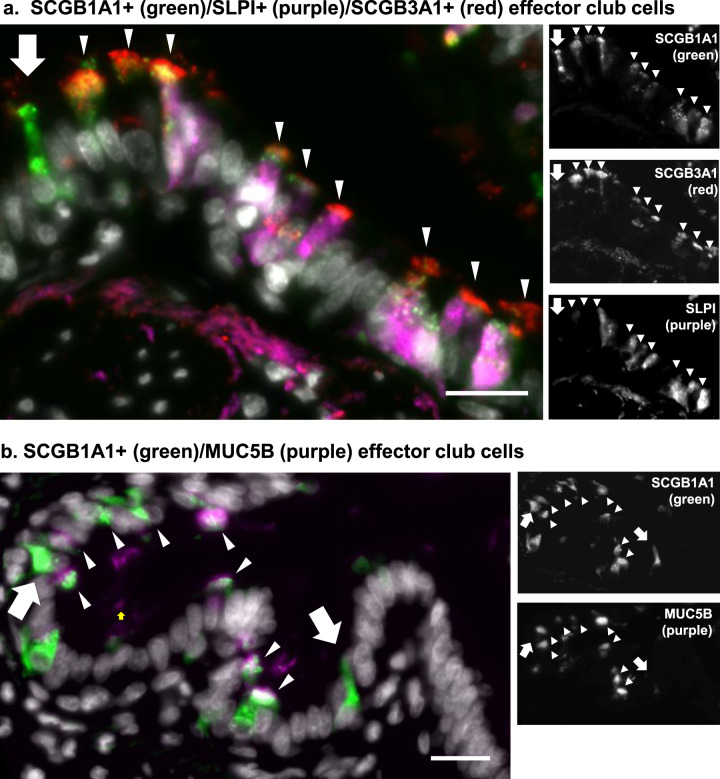
Immunohistochemistry assessment of club cell heterogeneity in the human lung. Airway in adult normal nonsmoker human lung was analyzed for club cell subtypes. Club cells were identified as SCGB1A1^+^ cells (green) and subsets were identified using various markers in the single cell sequencing. **a** Secretoglobin family 3A member 1 (SCGB3A1) (red) and secretory leukocyte protease inhibitor (SLPI) (purple). **b** SCGB1A1 (green), mucin 5B (MUC5B; purple). Club cells that only express SCGB1A1 but no secondary markers are denoted with a white arrow. Club cells expressing multiple markers are denoted with a white arrowhead. Scale bar = 20 μm.

Immunofluorescence histologic staining of nonsmoker human airways validated the concept of non-uniformity of the club cell population. While a majority of the club cells were positive for SLPI, SCGB3A1, and SCGB1A1, there were cells that were SCGB1A1-positive and SLPI-negative and SCGB3A1-negative (Fig. 2a). Similarly, a majority of club cells were SCGB1A1-positive and MUC5B-positive, though some were SCGB1A1-positive and MUC5B-negative (Fig. 2b). These data were consistent with a finding of heterogeneity among club cells. At this point, it is important to note that further discussion of club cell subsets will utilize the perspective of describing differential expression of genes among the club cell subclusters, but all of the cells in the ensuing analysis were initially clustered with club cells in the original unsupervised assessment of expression levels of ~30,000 genes in a total of over 11,000 cells (Supplementary Table 4).

### Club cell 1 is the progenitor club cell population

**Fig. 3:**
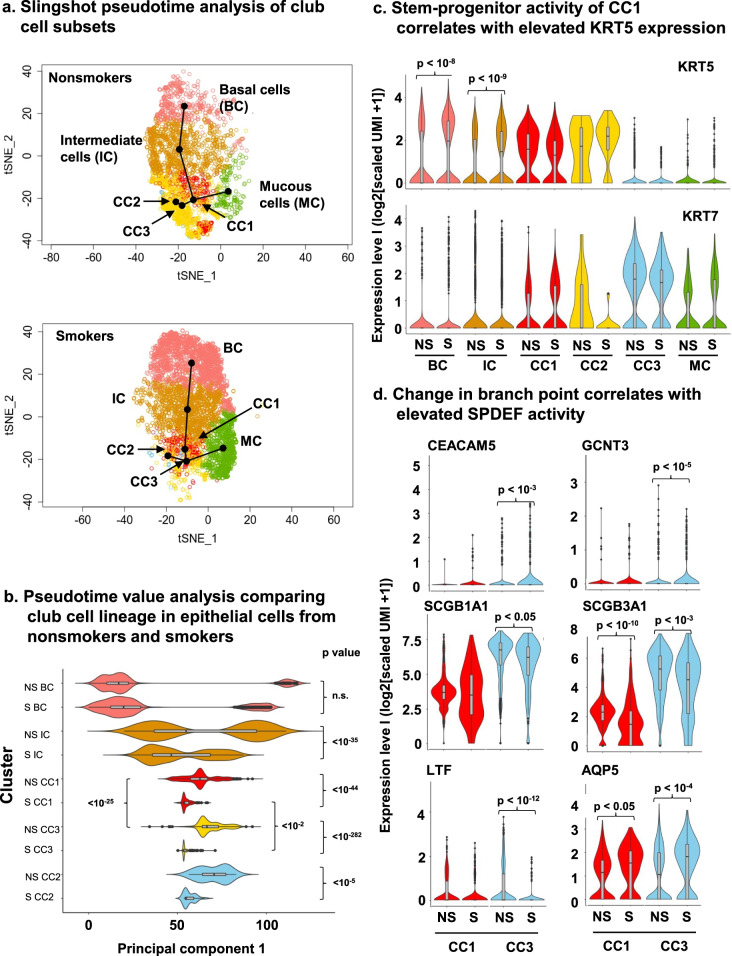
Pseudotime analysis of nonsmoker secretory cell differentiation. **a** Assessment of epithelial cell differentiation using Slingshot pseudotime value. Shown are epithelial cells differentiating in the secretory pathway including basal cells (BC, pink), intermediate cells (IC, orange), the progenitor club cell cluster (CC1, red), the effector club cell cluster (CC3, yellow), the proliferating club cell cluster (CC2, blue) and mucous cells (MC, green) for nonsmokers (NS) and smokers (S) plotted separately. Superimposed on each tSNE plot, the Slingshot pseudotime analysis shows the path of differentiation assuming basal cells as a starting point. **b** Pseudotime analysis. The extent of differentiation from the starting point was evaluated using a pseudotime analysis separately for nonsmokers (NS) and smokers (S). The results for each cluster have been plotted to enable pairwise comparisons. Significance of differences in the differentiation of each cluster is indicated by the *p* value to the right of the graph. *p* values were determined using a *t* test applied to the pseudotime values extracted by Slingshot. **c** Stem-progenitor activity of CC1 correlates with elevated KRT5 expression. Expression levels measured by the number of unique molecular identifiers (UMI) detected per cell during single cell RNA sequencing were plotted for KRT5 and KRT7 as a function of cluster for NS and S. Significant differences between NS and S are indicated by *p* values. Note that KRT5 levels in CC1 in NS have a mean that is greater than KRT5 expression in IC. KRT5 levels are considered a marker of stem-progenitor cells and are commonly thought to steadily fall as differentiation proceeds (see Supplementary Fig. 3). Expression of high levels of KRT5 in CC1 support CC1 as a branch point in the trajectory. At the same time, KRT7, a marker of club cells (see Supplementary Fig. 3), is clearly elevated in CC1 confirming the identity of CC1 as club cells rather than basal cells. **d** Change in branch point in smokers correlates with elevated SPDEF activity. Significant changes in expression levels of genes known to be up- or down-regulated by SPDEF were assessed in CC1 and CC3. Up-regulated genes included CEACAM5 and GCNT3. Down-regulated genes included SCGB1A1, SCGB3A1, LTF, and AQP5. Note that AQP5 is also up-regulated by oxidant stress such as exposure to cigarette smoke, potentially explaining the lack of down-regulation by smoking in CC1 and CC3. Significance of difference for panels **c** and **d** was determined based on the combined conditions of an adjusted *p* value < 0.05 (Bonferroni corrected) and a log fold change > 0.25. Violin plots in panels **b**–**d** include box-and-whisker plots showing the mean, 2nd and 3rd quartiles (box), range from minimum to maximum (whiskers), and outliers (dots).

The most common differentially expressed genes in club cell subpopulation 1 included KRT5 and KRT15, two basal cell-associated genes (Table 2). Club cells are normally described as KRT5 negative due to the very low level of detection of this gene (<1.2 UMI) in the population^31^. However, subclustering identified a group of club cells with higher levels of KRT5 expression, 1.3 log-fold relative to the remainder of the club cell population and detected in a high percentage of the cluster 1 cells (67%; Table 1). The presence of basal cell markers in SCGB1A1-expressing cells suggests that this population may have a genetic profile more similar to basal cells and might retain some degree of multipotency. To address this hypothesis more directly, the three club cell subsets were included in a Slingshot pseudotime analysis along with the other epithelial cells in the secretory lineage, including basal cell, intermediate cells, and mucous cells. A Slingshot pseudotime analysis of the entire nonsmoker secretory cell lineage showed that both club and mucous cells arose from basal cells through intermediate cells, and that the final populations of club cells and mucous cells resulted from a branch point centered on the progenitor subcluster CC1 (Fig. 3a). Surprisingly, the pseudotime analysis of smoker epithelial cells showed that the branch point for differentiation of smokers arose from the effector club cell subcluster CC3 rather than progenitor club cell subcluster CC1 (Fig. 3a). The identification of CC1 as a branch point is consistent with the proposed role of progenitor club cells as retaining some elements of a stem cell. Surprisingly, the Slingshot pseudotime analysis indicated that the branch point in smokers occurred at a different site, namely, at the effector club cell (subcluster 3). At a minimum, the result identifies normal cell differentiation pathways as a casualty of cigarette smoking, but it also provides a potential explanation for the observation that the small airways of smokers contain a reduced number of club cells and an elevated number of mucous cells^30^.

**Table 2:** Differentially expressed genes in progenitor vs. effector club cells^a^.

Progenitor club cells (cluster CC1)			Effector club cells (cluster CC3)		
Gene	Average log fold-change^b^	Corrected *p* value^c^	Gene	Average log fold-change^b^	Corrected *p* value^c^
KRT5^d^	1.52	1.50 × 10^−103^	PIGR	2.15	3.80 × 10^−100^
RPLP1	0.76	7.40 × 10^−102^	WFDC2	1.27	4.80 × 10^−97^
MIR205HG	1.34	1.50 × 10^−91^	SCGB3A1	2.50	6.70 × 10^−96^
AC016739.2	1.38	9.00 × 10^−91^	SLPI	1.17	1.60 × 10^−94^
RP11-234A1.1	1.21	1.60 × 10^−88^	SCGB1A1	2.04	2.30 × 10^−92^
S100A2	1.29	6.10 × 10^−88^	LCN2	1.70	7.30 ×10^−80^
RPS18	0.83	1.90 × 10^−86^	C3	1.60	2.90 × 10^−77^
RPL13P12	0.93	1.00 × 10^−82^	CP	1.17	7.60 × 10^−67^
MT-CO3	0.57	4.90 × 10^−78^	MUC1	1.42	2.40 × 10^−61^
CTD-3035D6.1	1.12	1.70 × 10^−77^	XBP1	1.30	5.50 × 10^−55^
RPL41	0.76	1.90 × 10^−76^	KRT7	0.99	1.70 × 10^−40^
RPS2	0.79	3.90 × 10^−76^	BPIFB1	2.29	7.50 × 10^−40^
MT-ND2	0.80	9.40 × 10^−74^	AGR2	0.74	1.80 × 10^−39^
RPL26	0.79	3.50 × 10^−73^	CD55	1.13	9.90 × 10^−37^
RPS27	0.72	4.10 × 10^−73^	CYP2F1	0.98	9.70 × 10^−34^
RPL10	0.76	1.60 × 10^−70^	S100P	1.39	2.10 × 10^−33^
RPL23	0.65	6.20 × 10^−69^	STEAP4	1.13	8.90 × 10^−33^
RPS6	0.52	9.10 × 10^−67^	VMO1	1.24	1.70 × 10^−31^
RPL28	0.66	3.90 × 10^−65^	NR4A1	1.20	4.10 × 10^−30^
MT-ND3	0.67	3.40 × 10^−64^	CEACAM6	1.22	9.20 × 10^−29^
RPL39	0.77	6.20 × 10^−64^	CYP2B7P	0.77	8.50 × 10^−28^
DST	1.22	8.50 × 10^−64^	TMC5	0.98	2.50 × 10^−26^
RPS15AP1	0.96	2.30 × 10^−63^	MUC5B	1.78	6.80 × 10^−26^
RPL13	0.47	1.00 × 10^−62^	FAM3D	0.83	3.60 × 10^−25^
RPS23	0.68	1.20 × 10^−62^	ELF3	0.66	3.20 × 10^−24^

^a^For each club cell subset, the 25 genes exhibiting the highest degree of upregulation compared to the other club cell subset are listed.

^b^Average fold change in comparison between these two groups.

^c^Bonferroni corrected *p* value.

^d^KRT5 is significantly increased in progenitor subcluster CC1, but KRT5 levels averaged across all cells in the larger combined club cell cluster are lower than KRT5 levels detected in basal cells and intermediate cells; see Supplementary Fig. 3.

To gain further insight into the shift in the branch point for mucous cell differentiation, an analysis of pseudotime value driven by slingshot analysis was conducted examining the club cell and mucous cell lineages separately (Fig. 3b). When comparing basal cells from nonsmokers and smokers in the club cell lineage, no difference was observed in pseudotime values (*p* > 0.05). However, for all other clusters, the pseudotime analysis indicated that cells in smoker epithelia lagged significantly behind nonsmoker epithelia in terms of differentiation. The most significant differences, based on *p* values comparing nonsmoker vs. smoker differentiation were observed in club cell subclusters 1 and 3. Of interest, nonsmoker club cell subclusters 1 and 3 clearly occupied distinct differentiation states (*p* < 10^−25^) while the difference between smoker club cell subclusters 1 and 3 barely reached significance (*p* < 10^−2^), further suggesting that the shift in the Slingshot pseudotime trajectory was due to a failure of club cell progenitors to completely differentiate into effector club cells, potentially leaving them with the stem properties of the club cell subcluster 1 cells and depleting detectable levels of club cells.

The Slingshot pseudotime trajectory showed that the branch point for mucous cell differentiation changed from CC1 to CC3 in smokers. Mucous differentiation is dependent on the activity of transcription factor SPDEF. A survey of the gene expression for 21 genes known to be influenced by SPDEF (genes upregulated by SPDEF: MUC5A, MUC16, AGR2, GCNT1, GCNT2, GCNT3, GALNT2, GALNT4, GALNT7, GALNT12, CEACAM5; genes downregulated by SPDEF: TTF1, FOXA2, SCGB1A1, SCGB3A1, AQP5, SCNN1B, SCNN1G, LTF)^32,33^ yielded significant smoking-induced changes in six genes (Fig. 3d). SPDEF-upregulated genes, CEACAM5 and GCNT3, showed significantly elevated expression in smokers compared to nonsmokers in CC3, but not in CC1. A set of three genes known to be transcriptionally inhibited by SPDEF, SCGB1A1, SCGB3A1, and LTF, were significantly down-regulated in CC3 with only one gene (SCGB1A1) being significantly down-regulated in CC1. One gene that was predicted to decrease with elevated SPDEF activity, AQP5, showed elevated expression in CC3 and CC1, likely a consequence of the reported oxidative stress response property not shared by the other SPDEF-responsive genes^34^.

### Club cell heterogeneity appears over time

**Fig. 4:**
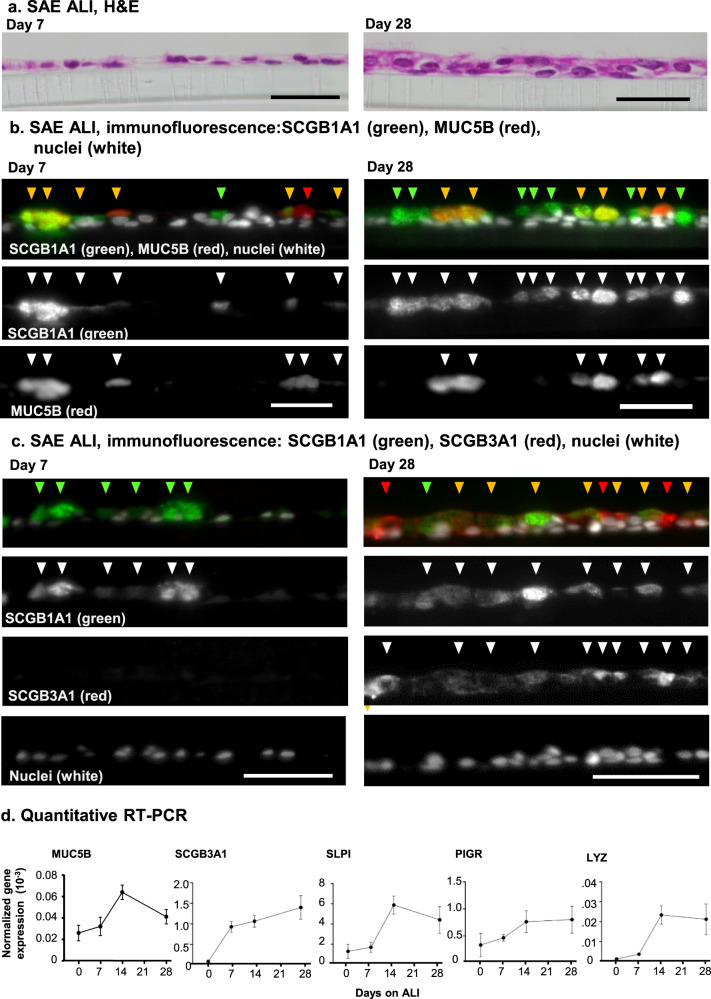
Heterogeneity during differentiation of club cells. Nonsmoker primary small airway epithelium (SAE) basal cells were cultured in air–liquid interface (ALI) culture for 28 days. **a** Morphology of the cultures at day 7 and 28. Shown are cross sections of the ALI culture, hematoxylin and eosin stain. **b**, **c** Multicolor immunofluorescence assessment of club cell subtypes. All club cells were identified using SCGB1A1 marker (green); effector club cells were further identified by the co-expression of MUC5B (panel **b** red) or SCGB3A1 (panel **c** red). DAPI identifies the nucleus of all cells. The number of days after establishment of the air liquid interface is noted. Green arrowheads mark cells that are SCGB1A1^+^ club cells that lack other markers. Orange arrowheads mark cells that are positive for SCGB1A1 and either MUC5B or SCGB3A1. Red arrowheads mark cells that are positive for MUC5B or SCGB3A1, but express little if any SCGB1A1. Scale bars are 50 μm. **d** Gene expression on ALI over time. TaqMan probes for genes enriched in effector club cells were assessed by qPCR and normalized to an 18S rRNA control. Probes were tested at days 0, 7, 14, and 28 of SAE nonsmoker cells differentiated in ALI. Error bars represent standard deviation among three different ALI cultures. Genes assessed for expression include MUC5B, SCGB1A1, SLPI, PIGR, and LYZ.

The concept of varied but related club cell differentiation states was further assessed using SAE basal stem/progenitor cells isolated from nonsmokers differentiating on air-liquid interface (ALI) culture over 28 days. During this differentiation period, basal cells developed from a monolayer to a differentiated epithelium consisting of multiple cell types based on morphology and ciliation (Fig. 4a). An early time point (day 7) and a differentiated end point (day 28) were analyzed by immunofluorescence staining using the club cell marker SCGB1A1 and secondary markers found in the club cell subpopulations (Table 2). At early stages of differentiation, MUC5B, a gel forming mucin, was apparent in both SCGB1A1^+^ and SCGB1A1^−^ cells. By day 28, while proportions of MUC5B and SCGB1A1 varied, all MUC5B cells had some level of SCGB1A1 expression. In contrast, cells that were SCGB1A1^+^ and MUC5B^−^ were clearly evident at day 28, suggesting that a population of SCGB1A1^+^ and MUC5B^−^ cells had emerged (Fig. 4b). SCGB3A1, a putative cytokine known to be expressed in club cells, showed a different timing of expression. At day 7, SCGB3A1 was barely detectable while SCGB1A1 club cells were already present. By day 28, multiple club cells expressed both SCGB1A1 and SCGB3A1 (Fig. 4c). Taqman RTqPCR analysis of MUC5B and SCGB3A1 corroborated the timing of the protein expression analysis, showing increased expression of theses markers over time, leveling off around day 28 when cells were fully differentiated (Fig. 4d).

### GAGE functional analysis

**Fig. 5:**
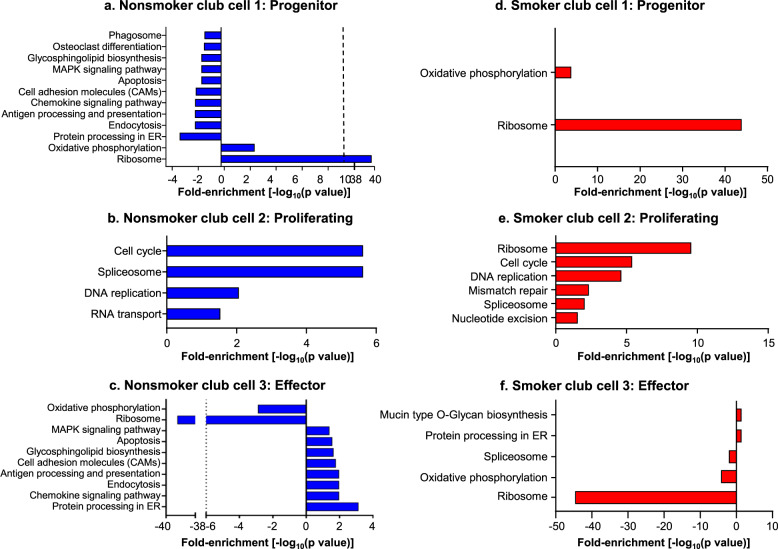
Different functions of the club cell subpopulations predicted using GAGE analysis in nonsmoker and smoker club cell subclusters. Shown are the enriched pathways in the three club cell populations of nonsmokers (**a**–**c**) and smokers (**d**–**f**). The Benjamini*–*Hochberg corrected *p* value indicating the significance of the relative contributions from each pathway are graphed using the negative of the logarithm of the corrected *p* value. Club cell subsets 1–3 were named according to their enrichment profile. **a** Nonsmoker progenitor club cells. **b** Nonsmoker proliferating club cells. **c** Nonsmoker effector club cells. **d** Smoker progenitor club cell. **e** Smoker proliferating club cells. **f** Smoker effector club cells. To enable a direct comparison of the effect of smoking on effector club cells, the GAGE analyses for nonsmoker and smoker effector club cells have been plotted together in Supplementary Fig. 4.

To define potential functional output of the three club cell subsets, GAGE was employed to examine biological processes enriched in the gene expression profiles of each subset^35^. Pathway analysis identified that club cell subcluster 1 was enriched in metabolic processes and ribosome biosynthesis, with minimal transcripts related to defense signaling and endocytic pathways, suggesting a metabolically active club cell (Fig. 5a). Club cell subcluster 2 was highly enriched in mitotic and cell cycle-related genes and was thus termed “proliferating club cells” (Fig. 5b). In contrast, club cell 3 was enriched in genes related to protein processing in the endoplasmic reticulum (ER), chemokine signaling, and antigen processing and presentation, with a relative reduction in ribosome and oxidative phosphorylation-related transcripts (Fig. 5c). Together with the differentiation analysis and expression of key functional genes, the superimposition of the functional pathway analysis led to the conclusion that club cell subcluster 1 represents “progenitor club cells” and club cell subcluster 3 represents “effector club cells”. To substantiate the effector club cell title, known club cell defense signatures were compared to the average gene enrichment per nonsmoker club cell subset, identifying an enriched defense gene signature in the effector club cells (Supplementary Table 5)^36^.

Knowing that the progenitor and effector club cell are furthest apart in gene-enrichment categories (Fig. 5a–c), comparison was made to determine the extent of the difference between these two divergent subclusters. Over 900 differentially expressed genes were identified between the two club cell subtypes (not shown). These differences were further supported by the finding of enrichment of Gene Ontology (GO) term biological processes including cell–cell adhesion, ATP biosynthesis, and ribosomal processing within the club cell 1 subset of cells, while subset 3 (effector club cells) was enriched in immunological function, response to lipopolysaccharide, and inflammatory response (Supplementary Table 5).

### Smoking alters club cell RNA signatures

A previous study from our laboratory suggested that chronic obstructive pulmonary disease (COPD) is an SAE club cell-deficient state^37–45^. Cigarette smoking is the most common risk factor for COPD leading to the hypothesis that smoking could result in disease-relevant alterations in club cell subpopulations. To address this hypothesis, mRNA signatures and enriched biological processes in smoker club cell subsets were analyzed (Fig. 5). While pathways enriched in progenitor and proliferating club cells obtained from smokers were similar to those of nonsmokers (Fig. 5d, e), there was a significant decrease in the club cell 3 defense-related functions (Fig. 5f). Furthermore, there were less enriched pathways in the progenitor and effector smoker club cells, with only two processes upregulated in the effector population, mucin type O glycan biosynthesis and protein processing in the ER.

**Fig. 6:**
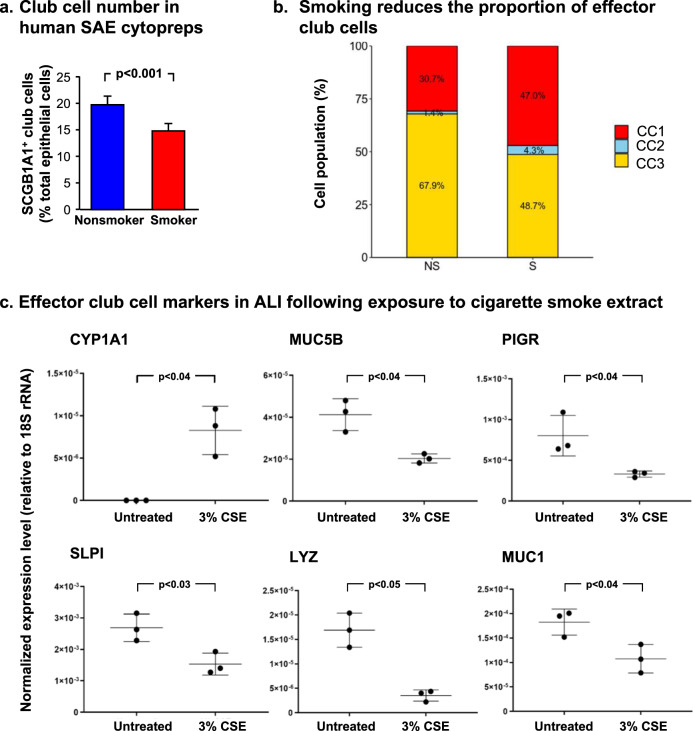
Reduction in the proportion of the of effector club cell subset in smokers. **a** Numbers of club cells in smokers vs. nonsmokers. Club cells from nonsmoker and smoker human small airway epithelium (SAE) cytopreps, identified by SCGB1A1^+^ immunostaining and absence of KRT5, were quantified and compared against total cells, quantified by DAPI staining, per cytoprep. Three samples of each phenotype were evaluated by a blinded observer, with over 500 total cells per sample; plots show mean ± standard error. **b** Proportion of club cells exhibiting characteristics of subclusters 1 (progenitor), 2 (proliferative), and 3 (effector) in the population of club cells derived from nonsmokers and smokers. **c** Effect of cigarette smoke extract (CSE) exposure on differentiation of small airway epithelial club cells. Exposure of basal cells differentiated on air liquid interface (ALI) to cigarette smoke extract (3% Marlboro Red) led to a decrease in defense-related transcript in cells: CYP1A1, positive control demonstrating exposure to cigarette smoke extract; MUC5B; PIGR; SLPI; LYZ; and MUC1. Values are expressed as normalized expression relative to 18S rRNA. Each point represents one well of an experiment done in triplicate; plot shows mean ± standard deviation; *p* values are from a two-sided unequal variance Student’s *t*-test. All data is from ALI day 28.

To examine the effects of smoking on each club cell subset, differentially expressed genes were analyzed between nonsmokers and smokers (Table 3). This analysis uncovered multiple genes dysregulated as a result of smoking for both the progenitor and effector clusters. As suggested in the GO term-enrichment analyses, known defense-related genes were significantly decreased in effector club cells of smokers (Supplementary Table 6). Furthermore, in progenitor club cells, there was a significant increase in the expression of genes known to be increased in smokers, such as FOS/JUN, as well as CYP1B1. Interestingly, proliferating club cells had no smoking-related differentially expressed genes (Table 3). Enrichment of host defense genes in subcluster 3 club cells is significantly decreased in smokers compared with nonsmokers (Figs. 5c and 6c). Direct comparison of nonsmoker and smoker effector club cell transcript confirmed the initial analysis and suggests that with exception of mucin type O-glycan biosynthesis and ER processing, expression of all other defense-related pathways was decreased (Supplementary Fig. 1).

**Table 3:** Smoking-related reprogramming of small airway epithelium club cells.

Progenitor club cells (CC1)						Effector club cells (CC3)					
Down-regulated in smokers			Up-regulated in smokers			Down-regulated in smokers			Up-regulated in smokers		
Gene	Average log fold-change^a^	pBon^b^	Gene	Average log fold-change^a^	pBon^b^	Gene	Average log fold-change^a^	pBon^b^	Gene	Average log fold-change^a^	pBon^b^
RP11-692N5.2	−1.30	1.00 × 10^−25^	RPS4Y1	1.32	1.30 × 10^−65^	XIST	−2.05	2.80 × 10^−79^	RPS4Y1	1.04	1.60 × 10^−87^
RPL37P2	−1.04	3.10 × 10^−24^	TXLNGY	0.74	1.60 × 10^−30^	SLPI	−0.59	2.50 × 10^−24^	DDX3Y	0.59	5.10 × 10^−39^
MTCO1P40	−1.43	6.90 × 10^−24^	ALDH3A1	0.81	3.60 × 10^−20^	C3	−0.89	3.20 × 10^−24^	TXLNGY	0.58	1.10 × 10^−36^
CTD-2287O16.1	−0.89	2.60 × 10^−21^	PRKY	0.49	1.80 × 10^−16^	MUC5B	−1.38	4.40 × 10^−13^	ALDH3A1	0.92	2.60 × 10^−32^
RPS20P24	−1.04	2.70 × 10^−21^	EIF1AY	0.41	1.10 × 10^−15^	LTF	−0.98	6.40 × 10^−13^	CYP1B1	0.80	1.90 × 10^−26^
RP11-777B9.5	−1.41	3.20 × 10^−20^	DDX3Y	0.47	2.60 × 10^−15^	LCN2	−0.58	1.90 × 10^−10^	NQO1	0.60	2.80 × 10^−24^
RP11-365F18.1	−0.87	1.70 × 10^−18^	CYP1B1	0.56	4.40 × 10^−14^	RP11-692N5.2	−0.60	2.00 × 10^−08^	EIF1AY	0.35	1.50 × 10^−22^
TSIX	−0.70	2.00 × 10^−18^	JUN	0.64	8.60 × 10^−13^	TNFAIP2	−0.78	3.20 × 10^−08^	PRKY	0.38	6.60 × 10^−20^
RPL21P1	−1.11	2.60 × 10^−18^	USP9Y	0.34	1.40 × 10^−09^	TSIX	−0.53	5.50 × 10^−08^	USP9Y	0.32	1.80 × 10^−18^
RPLP1P6	−1.24	4.90 × 10^−18^	SERPINB1	0.70	3.00 × 10^−09^	PROM1	−0.67	4.70 × 10^−07^	UTY	0.26	2.10 × 10^−18^
RPL30P4	−1.09	5.50 × 10^−18^	SFRP2	0.29	3.40 × 10^−09^	LYPD2	−0.87	6.00 × 10^−07^	MSLN	0.60	2.10 × 10^−15^
RPS29P5	−0.85	2.30 × 10^−17^	FTL	0.45	3.70 × 10^−09^	PIGR	−0.47	2.40 × 10^−06^	IER3	0.74	9.10 × 10^−15^
RPS19P1	−0.84	7.90 × 10^−17^	MTATP6P1	0.35	2.40 × 10^−08^	RPS24	−0.27	6.90 × 10^−06^	SFRP2	0.26	1.10 × 10^−14^
RPL31P12	−0.79	1.10 × 10^−15^	TSPAN1	0.43	2.60 × 10^−08^	RP11-355F16.1	−0.59	7.80 × 10^−06^	TTTY15	0.26	1.10 × 10^−14^
RP11-832N8.1	−0.81	3.90 × 10^−15^	KRT18	0.41	7.60 × 10^−08^	RPS4X	−0.31	4.70 × 10^−05^	RP4-765C7.2	0.68	1.10 × 10^−14^
RPS27AP11	−0.82	2.50 × 10^−14^	LY6D	0.44	1.00 × 10^−07^	SCGB3A1	−0.34	2.60 × 10^−04^	MTATP6P1	0.59	1.40 × 10^−14^
RPS12P26	−0.87	5.40 × 10^−14^	UTY	0.28	1.00 × 10^−07^	RPS29	−0.28	4.80 × 10^−04^	H19	0.68	4.50 × 10^−14^
RP11-360D2.2	−0.70	6.20 × 10^−14^	FTH1	0.28	1.50 × 10^−07^	PI3	−0.45	3.40 × 10^−03^	KRT8	0.58	4.90 × 10^−14^
PABPC1P3	−0.95	8.90 × 10^−14^	H19	0.64	1.70 × 10^−07^	GDA	−0.45	4.80 × 10^−03^	AKR1C2	0.58	5.10 × 10^−14^
RP11-571F15.3	−0.89	1.20 × 10^−13^	NR4A1	0.61	6.90 × 10^−07^	TMEM45A	−0.56	1.60 × 10^−02^	TSPAN1	0.45	6.20 × 10^−14^
MTRNR2L3	−1.09	1.30 × 10^−13^	BRCC3	0.48	7.10 × 10^−07^	SCGB1A1	−0.27	2.70 × 10^−02^	S100A6	0.34	1.70 × 10^−13^
RPL18P11	−0.59	1.60 × 10^−13^	CHP2	0.40	2.00 × 10^−06^	RPL21P1	−0.41	3.00 × 10^−02^	TMSB4X	0.45	5.10 ×10^−13^
RP11-20O24.1	−0.71	2.00 × 10^−13^	ATF3	0.72	9.70 × 10^−06^	RHOV	−0.45	4.50 × 10^−02^	NTS	1.01	1.40 × 10^−12^
RP11-392P7.1	−0.74	2.90 × 10^−13^	RPLP0	0.26	1.30 × 10^−05^	RP11-777B9.5	−0.43	4.90 × 10^−02^	F3	0.56	1.80 × 10^−12^
RPL24P8	−0.71	4.30 × 10^−13^	LINC01436	0.38	2.10 × 10^−05^						
RPS19P7	−0.61	5.10 × 10^−13^	FOS	0.43	2.60 × 10^−05^						
RP11-40C6.2	−0.71	5.60 × 10^−13^	IER3	0.50	9.10 × 10^−05^						
RP11-122G18.7	−0.71	6.10 × 10^−13^	AKR1C2	0.50	1.10 × 10^−04^						
RPS8P3	−0.71	4.10 × 10^−12^	FOSB	0.53	2.10 × 10^−04^						

^a^Average fold changes between smokers and nonsmokers in that group of cells.

^b^pBon = Bonferonni corrected *p* values.

Unlike the reduction in defense function in effector club cells, genes associated with negative apoptotic regulation and redox processes were increased in the effector club cells of smokers. This includes significant upregulation of genes such as DUOX1, NQO1, PRDX1, ALDH3A1, and CYP1B1, known regulators of oxidative–reduction pathways^46–49^ (Table 3). IER3, CLDN7, KRT18, MIF, SQSTM1, and CEACAM5 were also increased in expression, suggesting an inability to effectively eliminate damaged club cells^50–56^ (Table 3).

### Club cell defense function is reduced by smoking

**Fig. 7:**
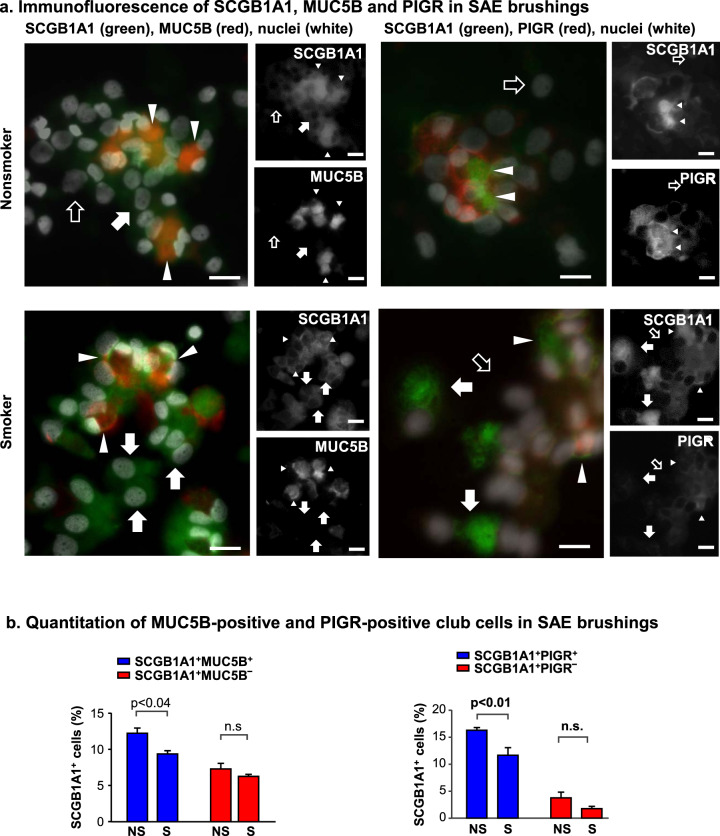
Smoking-induced, asymmetric decrease in club cell heterogeneity in small airway epithelial (SAE) cells brushed from the small airway of healthy nonsmokers or healthy smokers. **a** Immunofluorescence analysis of cytopreps containing club cells (SCGB1A1^+^) expressing the effector club cell genes PIGR or MUC5B. White arrowheads indicate dual-labeled cells. White arrows indicate SCGB1A1^+^ club cells that lack significant expression of a PIGR or MUC5B. Black arrows mark examples of SCGB1A1-negative non-club cells in the cytoprep. Bar = 20 µm. **b** Total number of dual labeled (SCGB1A1^+^PIGR^+^ or SCGB1A1^+^MUC5B^+^) cells, or SCGB1A1 single labeled cells were quantified by a blinded observer in nonsmokers and compared with smokers. Plot shows mean of three experiments ± standard error; *p* values are from a two-sided unequal variance Student’s *t*-test.

The GAGE analysis predicted a loss of effector club cell function in smokers. This finding was especially concerning in the context that smoking has previously been implicated in reducing the overall number of club cells in the airway^30^. This hypothesis was pursued by examining primary cells obtained from brushing, as well as cells differentiated in ALI culture in vitro. The proportion of club cells as a fraction of total cells recovered during bronchoscopic brushing of the human SAE significantly decreased in smokers, as quantified by the number of cells expressing SCGB1A1 identified by immunofluorescence staining of cytoprep samples (Fig. 6a). As a fraction of the overall club cells, subcluster 3 effector club cells represented a smaller fraction of the total population in smokers compared with nonsmokers (Fig. 6b). Together, the data indicate that smokers have fewer overall club cells and that those club cells contain a smaller percentage of effector club cells than nonsmokers. For independent confirmation of the loss of effector club cells following exposure to cigarette smoke, the ALI in vitro system was used to evaluate gene expression in nonsmoker basal cells differentiated in the presence or absence of cigarette smoke extract (CSE). Elevated expression of oxidant-sensitive CYP1A1 validated exposure to CSE. In support of the data obtained from the single cell RNA-seq analysis of nonsmoker and smoker club cells, the in vitro data demonstrated that key defense-related transcripts were also decreased with CSE exposure including MUC5B, PIGR, SLPI, LYZ, and MUC1 (Fig. 6c). To further assess this hypothesis, SAE samples obtained by bronchoscopic brushings from three healthy nonsmokers and three healthy smokers were analyzed by immunofluorescence for markers of effector club cells. To test for loss of effector club cells, the cluster 3 marker genes MUC5B and PIGR were used in conjunction with SCGB1A1 general club cell marker. Quantitative immunofluorescence uncovered an increase in the proportion of SCGB1A1^+^ cells that do not express either MUC5B or PIGR in smokers (Fig. 7). This analysis suggests that smoking may asymmetrically affect club cell heterogeneity in the smoker SAE. This finding is consistent with the transcriptomic data indicating that smoking leads to a decrease in the effector club cell population.

## Discussion

Club cells, representing 20% of the human small airway epithelium, play a central role in SAE defense^9^. Identified by the highly expressed marker SCGB1A1, club cells were previously considered to be a homogeneous population of secretory epithelial cells^10,11^. Using single-cell RNA-sequencing data and unsupervised clustering, significant heterogeneity was uncovered within the club cell population. Differentially expressed gene analysis led to the identification of three club cell categories: progenitor, effector, and proliferating club cells, with a proposed differentiation pathway of the progenitor club cells → effector club cells → proliferating club cells. The proportions of these club cells are significantly altered by cigarette smoking, resulting in a decrease in the effector club cell population, consistent with the concept that smoking is associated with a decrease in lung defenses^57–59^.

The classification of subgroups of previously identified cell type is driven by the availability of new technology and the need to explain observed phenomena. Identification of epithelial cell subsets was previously limited by available techniques, primarily relating to microscopy. Although a high-resolution technique, the low throughput and difficulty in establishing quantitative comparisons using microscopy limited the ability of investigators to differentiate among biochemically and functionally distinct epithelial cell types. With the advent of single cell RNA transcriptome analysis combined with highly quantitative and objective bioinformatics methods, a set of transcriptomes from a complex epithelium can be broken down into subclusters with successively higher resolution analysis. When the clusters align with previously observed distinctions such as basal cells, ciliated cells, and secretory cells, the transcriptome methods are validated. As the “perplexity” of the tSNE analysis is increased, further resolution is possible. For example, secretory cells can be resolved as club cells highly expressing SCGB1A1, mucous cells highly expressing MUC5AC, or intermediate cells that have not yet adopted a clear secretory profile. There is a limit to the useful sub-clustering of cell types. At an extreme, every cell has a unique transcriptome and could be considered its own cluster. To define a new subcluster, the subcluster should be observed in tissue from multiple independent donors, exhibit unique physiology, and correspond to a known function that has been attributed to the larger parent cluster. The three subclusters of club cells described here fulfilled those three criteria. All three subclusters were observed in all of the study subjects. The clusters exhibited differential response to the stress of cigarette smoking. And, as discussed below, the literature contains observations that are best explained by the presence of subclusters revealed by unsupervised clustering at higher resolution.

Previous reports define club cells as a defense-related cell type^9,11,13,17,19–22^. In the present study, we found that just <2/3 of club cells are enriched in defense-related transcripts relative to other club cells, suggesting that club cells may have multiple functions in the SAE.

One possible function is as a progenitor cell. In this regard, the club cell subset 1 expressed genes associated with high metabolic activity. The identification of this subcluster as stem-progenitor-related was initially spurred by the relatively high expression of KRT5, a basal cell marker, among the cells of this subcluster. At lower resolution, KRT5 was observed to exhibit a progressively lower expression from basal cells to intermediate cells to club cells to mucous cells, but in the context of the analysis of club cell subclusters, the CC1 progenitor cluster has a higher level of KRT5 expression than the intermediate cell cluster that precedes it in the pseudotime trajectory. The cells in the CC1 progenitor club cell cluster were initially placed into the club cell cluster based on their overall gene expression, making them authentic club cells, but the subcluster analysis was able to find this group of authentic club cells that had distinguishing characteristics such as high KRT5 expression suggesting a stem-progenitor function. Consistent with the role of progenitor club cells having pluripotency, the Slingshot pseudotime trajectory analysis identified this cluster as the branch point for further differentiation of both progenitor club cells and mucous cells. Progenitor club cells have a significant overlap with metabolically active cell signatures with enrichment in genes such as ALDOA, LDHB, ENO1, COX7B and C, COX8A, ATP5G, and ALDH3A2^60–65^. This observation of elevated metabolic activity, paired with the expression of cytokeratins that are synonymous with progenitor cell types (KRT5, KRT15), could be indicative of retained stemness and a potential for multi-potency. Further studies focusing on the club cell subset isolation and in vitro differentiation potential will help to understand the enrichment of metabolic processes on the stem/progenitor function of the precursor club cells.

An examination of recent literature relating to epithelial cell differentiation concludes that human club cells, in addition to their distinct role in host defense, are also capable of further differentiation along both the secretory and ciliated cell pathways^66^. Ruiz Garcia et al. reported co-localization of KRT5 and KRT7 in the nasal epithelium in cells that clearly had a columnar morphology, consistent with the characteristics of progenitor CC1 cells described here^67^.

Effector club cells, unlike the proliferating and progenitor club cells, display marked enrichment in MUC5B, PIGR, CXCL1, CXCL6, LCN2, MUC1, BPIFB1, and CXCL17 anti-inflammatory and immune-related mRNA transcripts. Enriched in the majority of previously shown club cell transcripts^36^, subset 3 of club cells likely is focused on fulfilling the well-established role of club cells in host defense. Our analysis found that these effector club cells represent nearly 2/3 of the club cell population, highlighting the insight from single cell sequencing and subclustering analysis in order to separate out unique functions within a single cell type.

The phenomenon of proliferating club cells was observed by Boers et al.^68^. The data in the present study of club cell subset 2 is consistent with this data and shows that these club cells are a small, distinct portion of the population with unique identifying gene signatures. Furthermore, the data suggests that these cells are not the least differentiated of the club cell subset. The increase of this subtype in smokers leads to a possibility that these cells may be responding to unique environmental cues. This provides more evidence of club cells as a non-terminal cell state and having the potential to maintain homeostasis in the airway.

Other studies have reported heterogeneity in human club cell populations. Ruiz Garcia et al.^67^ examined human airway epithelia differentiated on ALI and found evidence for three club cell subsets. One of the three subsets (their CC2) shared properties with the effector club cell cluster in this report. An alignment between the other two subsets and clusters CC1 and CC3 here is not as straightforward. Nevertheless, the principle of heterogeneity among club cells is common to both studies.

The Slingshot pseudotime trajectory analysis gave important insights into the pathophysiology and targets of cigarette smoking. The Slingshot analysis showed that cigarette smoking imposes a global inhibition on club cell differentiation that is evident in intermediate cells and has a dramatic impact on all three club cell subclusters, essentially halting their differentiation early after club cell differentiation. Interestingly, differentiation proceeded far enough so that representatives of all three club cell subclusters were present in all three smoker subjects. The impact of cigarette smoking was further evident in the trajectory analysis indicating that CC3 effector club cells rather than CC1 progenitor club cells had become the branch point for mucous cell differentiation. Consistent with this observation, signaling pathways related to mucous cell-producing SPDEF were upregulated in CC3 effector cells and competing club cell-producing pathways (FOXA2, TTF-1) were downregulated. These findings are also consistent with a recent report that CSE, acting through Notch 3, induces SPDEF signaling and mucous cell differentiation^69^.

Identification of club cell clusters is important to our understanding of the response of the airway environmental and/or genetic pathophysiology. Cigarette smoking is the leading cause of COPD, a disease characterized by a decreased proportion of club cells in the SAE^30,37–43,45^. Consistent with this concept, our analysis found that smoking reduces club cell numbers, and, importantly, selectively reduces the proportion of effector club cells to less than half of the total number of club cells. Interestingly, only the effector club cell population with defense transcripts appears to be significantly decreased. Furthermore, direct comparison of the remaining smoker effector club cell transcriptome with nonsmokers shows a marked loss of defense-related transcripts, suggesting a potential decrease in defense function as a result of smoking. This includes the known club cell defense genes MUC5B, CXCL1, SCGB3A1, SLPI, and PIGR. For example, PIGR has been shown to be essential for airway defense function and a decrease in PIGR correlated directly with the severity of disease^70^. The Slingshot pseudotime trajectory analysis suggested that smoking induced a change in the branch point for mucous cell production from CC1 progenitor club cells to CC3 effector club cells. In addition to explaining the increase in mucous cells, this change would help to explain the depletion in effector club. Analysis of the SPDEF signaling pathway provides one potential set of targets to maintain CC3 effector club cell production in the airway.

## Methods

### Study population and sample acquisition

Subjects (three healthy nonsmokers and three healthy smokers with normal lung function) were recruited under a protocol approved by Weill Cornell Medical College Institutional Review Board and provided written informed consent. See Supplementary Table 1 for full demographic details. Gender and age have been separately evaluated as potential confounding factors in the dataset and have been ruled out as a source of bias (see Supplementary Tables 2, 3). All subjects underwent comprehensive screening, including medical history, physical examination, routine blood work, full pulmonary function testing, chest radiograph and urinary nicotine metabolite testing to confirm their smoking status prior to research bronchoscopy with brushing of the SAE (10th–12th generation airways) as previously described^36^.

### Single cell isolation, and single cell RNA sequencing

A single cell suspension of living cells from SAE samples was created by trypsinization, followed by selection of single living cells via flow cytometry with negative selection of dead cells using 4′,6-diamidino-2-phenylindole (DAPI). Live single cells were analyzed in the Weill Cornell Genomics Core Facility by the Drop-seq method^71^. Sequence from cDNA libraries was obtained using Illumina HiSeq 2500 (Illumina, San Diego, CA). Quality control data for the dataset are provided in Supplementary Table 4.

### Clustering analysis

A clustering algorithm for the six SAE samples was performed using Seurat, a developed R package for single cell analysis^72^. Raw digital expression matrices containing integer counts of number of transcripts for each gene in each cell were generated separately for each sequence using the McCarroll lab protocol^71,73^. Data was filtered based on the following criteria: (1) all genes evaluated were expressed in ≥10 cells and only cells with a minimum of 200 detected genes were retained for analysis; (2) cells expressing >10,000 or <200 unique genes were removed; and (3) cells with >25% mitochondrial genes were removed. Data was further processed and cell clusters identified as described by Zuo et al.^36^. Briefly, the clustering algorithm, *K*-nearest neighbor graph based on the Euclidean distance in principal component analysis (PCA) space was applied to group cells together iteratively leading to cells most similar to each other being clustered together. Eleven distinct clusters were identified and signature genes differentially expressed in each cluster were used to identify cluster cell types^36^.

### Subclustering of club cells

Club cells, defined as cells with high SCGB1A1 expression but low levels of KRT5 and MUC5AC expression [<1.2 unique molecular identifiers (UMI)] were analyzed separately from the total SAE. There were 1203 variably expressed genes in the club cell population of SCGCB1A1^+^KRT5^lo^MUC5AC‾ cells. Dimensional reduction PCA on variably expressed genes was applied. The clustering algorithm, *K*-nearest neighbor graph based in PCA space approach implemented in “FindClusters” function in Seurat package, was applied to find clusters in club cells yielding three unique club cell populations. To determine the marker genes for these subclusters, cells from each subcluster were compared to all other club cells using the Seurat “FindMarkers” function. Marker genes were required to have an average expression in the subcluster 0.25 log fold higher than the average expression in other club cell subclusters and were required to be expressed in ≥10% of cells in that subcluster. A Bonferroni correction was used to adjust *p* values. For visualization purposes, club cell subclusters were presented in tSNE plots.

### Cell trajectory

Single cell pseudotime trajectories were constructed with Slingshot. Slingshot uses preexisting clustering results to infer lineage tracing based on minimal spanning tree method^74^. Distance moved along the trajectory was determined, and pseudotime values were extracted using the slingPseudotime function in the Slingshot package. The pseudotime values were plotted based on a violin plot in R.

### Single cell statistical analysis

To identify signature genes in each subcluster, the expression of genes from each cluster were compared to the expression of genes from all cells of remaining clusters using the Seurat “FindAllMarkers” function, and markers were identified by the Wilcoxon rank sum test for single cell gene expression^75^. Gene marker criteria were: expression levels differed by at least 0.25 log-fold between the subject cluster and all comparison clusters; and the marker was expressed in ≥10% of cells the subcluster. Genes expressed below an average UMI of 1.2 were removed from analysis. Adjusted *p* values were calculated using a Bonferroni correction based on the total number of genes in the dataset. The generally applicable gene-set enrichment for pathway analysis (GAGE) package for R Version 2.36.0 was used for gene set enrichment analyses^35^.

To identify differentially expressed genes (DEGs) between smokers and nonsmokers, each cell type was evaluated independently and used Wilcoxon rank sum test as implemented in the Seurat FindMarkers. Genes were considered differentially expressed if the adjusted *p* value (Bonferroni correction) was <0.05 and log fold change was >0.25 for genes expressed at least in 10% of cells.

### Air–liquid interface (ALI) epithelial cell cultures

Basal cells collected from the small airway by brushing as described above were expanded on type IV collagen and frozen as previously described^36^ with the following modifications. Basal cells were expanded in PneumaCult ExPlus Basal medium (StemCell Technologies, Cambridge, MA) on polystyrene T75 flasks (Corning, Corning, NY) previously coated with a solution of 0.3 mg/mL collagen type IV from human placenta (Sigma-Aldrich, St. Louis, MO) for 1 h at 37 °C, washed with sterile PBS, and dried. To create a differentiated airway epithelial cell culture grown on ALI, basal cells were isolated using 0.05% trypsin (in 0.7 mM ethylenediaminetetraacetic acid EDTA (Gibco, ThermoFisher Scientific, Waltham, MA) and seeded at a density of 10^5^ basal cells on collagen IV-coated Costar Transwell inserts (6.5 mm, 0.4 µm; StemCell Technologies) with ExPlus complete growth medium in both the apical (0.1 mL) and basolateral (0.5 mL) chambers (ALI day −2). Cells were expanded for 2 days and then ExPlus complete growth medium was replaced with PneumaCult ALI-S maintenance medium (StemCell Technologies) in the lower chamber only with the apical chamber exposed to air (ALI day 0). Basal cells were allowed to differentiate in ALI culture with media freshly prepared and changed every 2 days. At day 14 through day 28, apical surfaces were washed with PBS (Gibco, ThermoFisher Scientific) once per week. Samples were collected at days 0, 7, 14, and 28 for analysis of mRNA and protein expression. A limited supply of the primary basal cells used for these studies have been retained and may be made available to qualified scientists by contacting the corresponding author.

### RTqPCR

Cells from three ALI transwells per time point were rinsed with PBS and collected in 1 mL of Trizol (ThermoFisher Scientific). Chloroform was utilized to precipitate the RNA and RNeasy kits (Qiagen, Germantown, MD) were used to elute RNA. Reverse transcriptase reactions were performed using Applied Biosystems TaqMan Reverse Transcription Reagents kit (Applied Biosystems by Life Technologies,) with 1 μg of RNA in 50 μL reaction. To assess the timing of expression of club cell-specific markers, the following probes (ThermoFisher Scientific) were utilized, including: 18S RNA (4310893E), MUC5B (Hs00861588_m1), SCGB1A1 (Hs00171092_m1), SCGB3A1 (Hs00369360_g1), LYZ (Hs00426232_m1), PIGR (Hs00922561_m1) and SLPI (Hs00268204_m1). All data was normalized to 18S RNA.

### Immunofluorescence assessment of protein expression

Paraffin-embedded normal human lung sections were obtained from BioMax (Rockville, MD) and ALI in vitro sections were embedded and sectioned by Histoserv (Germantown, MD). Immunofluorescence staining was performed on paraffin-embedded cross-sections and ALI sections. Freshly brushed SAE cells were fixed with 4% paraformaldehyde and permeabilized with 0.01% Triton X-100. All samples were blocked with 10% donkey serum prior to staining. The following primary antibodies were applied to samples overnight at 4 °C: rabbit polyclonal anti-human MUC5B (sc-20119; Santa Cruz; Santa Cruz, CA; 1:50 dilution); rabbit polyclonal anti-human PIGR (HPA012012; Sigma; affinity purified against the immunogen: 1:100 dilution); rabbit polycloncal anti-human SLPI (NBP1-89139; Novus Biologicals, Centennial, CO; affinity purified against immunogen; 1:100 dilution); mouse monoclonal anti-human SCGB3A1 (MAB27901; R&D, Minneapolis, MN; purified monoclonal antibody; purified from hybridoma; 1:50 dilution from 0.5 mg/mL solution); and rat monoclonal anti-human SCGB1A1 (MAB4218; R&D Systems; purified from hybridoma; 1:100 dilution from 0.5 mg/mL solution). Isotype matched non-specific IgG-negative controls included: rat IgG1 (R&D Systems), rabbit IgG, and mouse IgG (ThermoFisher Scientific). To visualize the antibody binding, the following secondary antibodies were used: Alexa Fluor 555 donkey anti-Mouse IgG (A-32773; Invitrogen), Alexa Fluor 488 donkey anti-rat IgG (A-21208, Invitrogen), and Alexa Fluor 647 donkey anti-rabbit IgG (A-31573; Invitrogen), all highly cross-adsorbed by the manufacturer and used at a 1:500 dilution from the stock concentration. The cells were counterstained with DAPI to identify cell nuclei and mounted using ProLong Gold antifade reagent (P36930; Invitrogen).

Immunofluorescence microscopy was performed using a Zeiss Axioplan microscope with a ×40 objective and captured with a Zeiss high-resolution monochrome camera. Images of lung cross sections were obtained with a Zeiss 880 confocal microscope using ×40 and ×60 objectives with oil immersion. Images were processed using Zen (Carl Zeiss Microscopy, White Plains, NY) and ImageJ software (https://imagej.net/Fiji). For quantitative assessment, images were acquired using fixed exposure settings optimized for each channel in a single data acquisition session. Prior to quantitation, a threshold was applied to images from each channel to create a minimum signal level constituting a positive signal. One or more marker channels were overlaid and combined with the DAPI channel to provide a count of total cells. The incidence of co-localization was scored by a blinded observer according to the presence of a positive pixel value in each channel in the space morphologically associated with a particular nucleus. Five fields from each of the three preparations were scored per condition. A minimum of one hundred cells per phenotype were assayed per immunofluorescence condition.

### Graphical analyses and statistics

All graphical analyses were done using GraphPad Prism Software version 8.1 (San Diego, CA). Two-tailed Student’s *t*-test with unequal variance was used for all in vitro statistical analyses. For the pseudotime analysis, a two-tailed *t* test function in R was used to compare the pseudotime values produced by Slingshot for each group of cells.

### Reporting summary

Further information on research design is available in the Nature Research Reporting Summary linked to this article.

## Supplementary information [Publisher: new figures have been uploaded; to conform to edits in text]

Reporting Summary

Supplementary Information

## Data Availability

The single cell RNA sequencing data in Figs. 1, 3, 5, 6 and S1–4 were originally published elsewhere^31^ and are associated with Gene Expression Omnibus (GEO) Accession Number GSE123405; the data associated with this paper have been re-submitted to GEO with Accession Number GSE155515.
