# Supplementary material for: Smoking shifts human small airway epithelium club cells toward a lesser differentiated population
Source: NPJ Genom Med. 2021 Sep 8;6:73. doi: 10.1038/s41525-021-00237-1 (PMC8426481; doi:10.1038/s41525-021-00237-1)
Supplement: Supplementary file 1 — Reporting Summary [file 41525_2021_237_MOESM1_ESM.pdf]

nature research

Corresponding author(s): Ronald G. Crystal

Last updated by author(s): Jun 29, 2021

# Reporting Summary

Nature Research wishes to improve the reproducibility of the work that we publish. This form provides structure for consistency and transparency in reporting. For further information on Nature Research policies, see our Editorial Policies and the Editorial Policy Checklist.

## Statistics

For all statistical analyses, confirm that the following items are present in the figure legend, table legend, main text, or Methods section.

| n/a | Confirmed | |
|---|---|---|
| ☐ | ☒ | The exact sample size (*n*) for each experimental group/condition, given as a discrete number and unit of measurement |
| ☐ | ☒ | A statement on whether measurements were taken from distinct samples or whether the same sample was measured repeatedly |
| ☐ | ☒ | The statistical test(s) used AND whether they are one- or two-sided<br>*Only common tests should be described solely by name; describe more complex techniques in the Methods section.* |
| ☒ | ☐ | A description of all covariates tested |
| ☐ | ☒ | A description of any assumptions or corrections, such as tests of normality and adjustment for multiple comparisons |
| ☒ | ☐ | A full description of the statistical parameters including central tendency (e.g. means) or other basic estimates (e.g. regression coefficient) AND variation (e.g. standard deviation) or associated estimates of uncertainty (e.g. confidence intervals) |
| ☒ | ☐ | For null hypothesis testing, the test statistic (e.g. *F*, *t*, *r*) with confidence intervals, effect sizes, degrees of freedom and *P* value noted<br>*Give P values as exact values whenever suitable.* |
| ☒ | ☐ | For Bayesian analysis, information on the choice of priors and Markov chain Monte Carlo settings |
| ☒ | ☐ | For hierarchical and complex designs, identification of the appropriate level for tests and full reporting of outcomes |
| ☒ | ☐ | Estimates of effect sizes (e.g. Cohen's *d*, Pearson's *r*), indicating how they were calculated |

*Our web collection on statistics for biologists contains articles on many of the points above.*

## Software and code

Policy information about availability of computer code

| | |
|---|---|
| Data collection | Details provided in Methods. No custom code was used. |
| Data analysis | Details provided in Methods. No custom code was used. |

For manuscripts utilizing custom algorithms or software that are central to the research but not yet described in published literature, software must be made available to editors and reviewers. We strongly encourage code deposition in a community repository (e.g. GitHub). See the Nature Research guidelines for submitting code & software for further information.

## Data

Policy information about availability of data

All manuscripts must include a data availability statement. This statement should provide the following information, where applicable:

- Accession codes, unique identifiers, or web links for publicly available datasets
- A list of figures that have associated raw data
- A description of any restrictions on data availability

The single cell RNA sequencing data in Figs 1, 3, 5, 6, and S1-4 were originally published elsewhere31 and are available in Gene Expression Omnibus (GEO) site with accession number: GSE155515 (https://www.ncbi.nlm.nih.gov/geo/query/acc.cgi?acc=GSE155515)31.

# Field-specific reporting

Please select the one below that is the best fit for your research. If you are not sure, read the appropriate sections before making your selection.

☒ Life sciences ☐ Behavioural & social sciences ☐ Ecological, evolutionary & environmental sciences

For a reference copy of the document with all sections, see nature.com/documents/nr-reporting-summary-flat.pdf

# Life sciences study design

All studies must disclose on these points even when the disclosure is negative.

| | |
|---|---|
| Sample size | A sample size of N = 6 provided statistically significant data. |
| Data exclusions | Single cell data for individual cells not meeting quality control requirements were excluded. |
| Replication | N for each experiment are provided in the text. |
| Randomization | The first 6 volunteers recruited were included in the study. No randomization occurred. |
| Blinding | Blinding was used in the assessment of immunofluorescence staining as described in the Methods. |

# Reporting for specific materials, systems and methods

We require information from authors about some types of materials, experimental systems and methods used in many studies. Here, indicate whether each material, system or method listed is relevant to your study. If you are not sure if a list item applies to your research, read the appropriate section before selecting a response.

## Materials & experimental systems

| n/a | Involved in the study | |
|---|---|---|
| ☐ | ☒ | Antibodies |
| ☒ | ☐ | Eukaryotic cell lines |
| ☒ | ☐ | Palaeontology and archaeology |
| ☒ | ☐ | Animals and other organisms |
| ☐ | ☒ | Human research participants |
| ☒ | ☐ | Clinical data |
| ☒ | ☐ | Dual use research of concern |

## Methods

| n/a | Involved in the study | |
|---|---|---|
| ☒ | ☐ | ChIP-seq |
| ☒ | ☐ | Flow cytometry |
| ☒ | ☐ | MRI-based neuroimaging |

## Antibodies

| | |
|---|---|
| Antibodies used | Clone numbers, manufacturer, method of purification, and dilution are provided in the Methods section. |
| Validation | Primary antibody staining was optimized by testing dilutions of antibodies compared with serum or non-specific antibody controls. Manufacturer's used orthogonal validation (molecular correlation or morphometry) to validate products used in this study. Where reagents were purified based on affinity for the target antigen, a note is added in the Methods section. Investigator validation of antibody staining occurred through examination of the unique morphologies produced in the staining patterns for each antigen. |

## Human research participants

Policy information about studies involving human research participants

| | |
|---|---|
| Population characteristics | See Supplemental Table I |
| Recruitment | Study volunteers were recruited using IRB-approved print, radio, online, and direct-mail advertising. |
| Ethics oversight | Weill Cornell Medicine Institutional Review Board |

Note that full information on the approval of the study protocol must also be provided in the manuscript.
