# Supplementary material for: Smoking shifts human small airway epithelium club cells toward a lesser differentiated population
Source: NPJ Genom Med. 2021 Sep 8;6:73. doi: 10.1038/s41525-021-00237-1 (PMC8426481; doi:10.1038/s41525-021-00237-1)

Supplementary Table 1. Demographics of Nonsmokers and Smokers[1]

Parameter	Nonsmokers	Smokers	p value
N	3	3	
Gender (M/F) [2]	0/3	3/0	p>0.1
Age (yr) [2]	26 ± 8	42 ± 14	p>0.1
Race (B/W/H/O)[3]	1/1/0/1	1/0/1/1	p>0.8
Body Mass Index	23 ± 4	23 ± 4	p>0.8
Smoking history			
Age of initiation	NA	20 ± 1	NA
Pack years	NA	12 ± 6	NA
Urine nicotine (ng/ml)[4]	0	844 ± 616	NA
Urine cotinine (ng/ml)[4]	0	936 ± 458	NA
Carboxyhemoglobin (%)	1.6 ± 0.1	2.3 ± 0.2	p>0.1
Pulmonary function parameters[5]			
FVC	115 ± 15	102 ± 13	p>0.5
FEV1	110 ± 12	100 ± 10	p>0.4
FEV1/FVC	83 ± 5	80 ± 4	p>0.4
DL_{CO}	89 ± 8	92 ± 10	p>0.1
TLC	107 ± 12	103 ± 6	p>0.7
Cough Score[6]	0.7 ± 0.6	1.3 ± 0.6	p>0.3
Sputum Score[6]	0.7 ± 0.6	1.3 ± 0.6	p>0.3
SAE cell differential (%)			
Epithelial	96.6 ± 1.1	98.9 ± 0.2	p>0.06
Inflammatory	3.4 ± 1.1	1.1 ± 0.2	p>0.06
Ciliated	59.9 ± 2.0	57.7 ± 9.9	p>0.7
Secretory	10.3 ± 2.5	9.6 ± 5.1	p>0.8
Undifferentiated	24.0 ± 0.7	29.8 ± 5.8	p>0.2
Basal	2.4 ± 1.0	1.8 ± 1.0	p>0.4

[1] Data are presented as mean ± standard deviation; p values of numeric parameters calculated using a 2-tailed Student's t-test; p value of categorical parameters calculated using a Chi-square test with with Yates correction due to the low n of samples; this table reproduced with permission from Zuo et al[1].

[2] See Supplemental Tables II and III for data on the impact of gender and age, respectively, on gene expression relative to smoking

[3] Abbreviations B=Black, W=White, H=Hispanic, O=Other, NA=not applicable; FVC - forced vital capacity, FEV1 - forced expiratory volume in 1 sec, TLC - total lung capacity, DL_{CO} - diffusing capacity of the lung for carbon monoxide, SAE=small airway epithelium.

[4] Undetectable urine nicotine <2 ng/ml; cotinine < 5 ng/ml.

[5] Pulmonary function testing parameters are given as % of predicted value with the exception of FEV1/FVC which is reported as % observed

[6] Cough and sputum score were each evaluated on a scale of 0-4: 0 = not at all; 1 = only with chest infections; 2 = a few days a month; 3 = several days a week; 4 - most days a week [2].

Supplementary Table 2. Effect of Gender *vs* Smoking on Small Airway Epithelium Gene Expression[1]

	Microarray[2]		RNA-Seq[3]	
**Comparison[1]**	**n of subjects[2]**	**n of genes[4]**	**n of subjects**	**n of genes[4]**
Nonsmoker M *vs* nonsmoker F	38 M *vs* 22 F	30	10 M *vs* 10 F	42
Smoker M *vs* smoker F	53 M *vs* 20 F	23	20 M *vs* 3 F	16
M *vs* F	91 M *vs* 42 F	40	30 M *vs* 13 F	68
Smokers *vs* nonsmokers	73 S *vs* 63 NS	3,408	23 S *vs* 20 NS	2,454

1 Analysis of previously acquired datasets; M = males, F = females, NS = healthy nonsmokers, S = asymptomatic smokers. This table reproduced with permission from Zuo et al[1].

2 Small airway epithelial samples processed on Affymetrix HG-U133 Plus 2.0 microarrays (Affymtrix); previously published (GEO accession # 77658).

3 Small airway epithelial samples processed on Illumina Hi Seq 2500 (Illumina); a subset of the samples has been previously published (GEO accession # 92661).

4 N of genes differentially expressed when comparing the groups on a genome-wide basis [in microarray: n=14,465 genes (present in at least 20% of the samples in each group , one probe per gene, chosen based on Affymetrix specificity and sensitivity scores); in RNA-Seq: n=16,140 genes (FPKM >0.125)]; p value corrected for multiple testing (Benjamini-Hochberg) <0.05 considered significant.

Supplementary Table 3. Effect of Age *vs* Smoking on Small Airway Epithelium Gene Expression[1]

	Microarray[2]		RNA-Seq[3]	
**Comparison[1]**	**n of subjects[2]**	**n of genes[4]**	**n of subjects**	**n of genes[4]**
NS old *vs* NS young	31 old *vs* 29 young	0	8 old *vs* 12 young	0
S old *vs* S young	55 old *vs* 18 young	2	15 old *vs* 8 young	1
Old *vs* young	86 old *vs* 47 young	301	23 old *vs* 20 young	0
S *vs* NS	73 S *vs* 63 NS	3,408	23 S *vs* 20 NS	2,454

[1] Analysis of previously acquired datasets; old ≥ 40 years old, young = 18-39 years old, NS = healthy nonsmokers, S = healthy smokers.

[2] Small airway epithelial samples processed on Affymetrix HG-U133 Plus 2.0 microarrays (Affymtrix); previously published (GEO accession # 77658).

[3] Small airway epithelial samples processed on Illumina Hi Seq 2500 (Illumina); a subset of the samples has been previously published (GEO accession # 92661).

[4] N of genes differentially expressed when comparing the groups on a genome-wide basis [in microarray: n=14,465 genes (present in at least 20% of the samples in each group , one probe per gene, chosen based on Affymetrix specificity and sensitivity scores); in RNA-Seq: n=16,140 genes (FPKM >0.125)]; p value corrected for multiple testing (Benjamini-Hochberg) <0.05 considered significant.

Supplementary Table 4. Quality Control for the Single-cell RNA-sequencing Data in Each Individual[1]

Pheno-type	Total reads	Mean reads/cell	Number of gene detected	Median genes/cell	Median UMI count/cell
Nonsmoker-1	112,793,786	91,405	29,816	736	1,390
Nonsmoker-2	131,807,942	107,686	31,800	896	1,517
Nonsmoker-3	67,651,975	29,842	30,250	769	1,259
Smoker-1	113,288,982	43,690	31,544	816	1,393
Smoker-2	47,156,371	23,472	28,539	830	1,465
Smoker-3	128,445,800	54,082	31,840	727	1,247

[1]Table reflects quality control data after applying filters during processing; UMI = unique molecular identifiers; reproduced with permission from Zuo et al[1]

Supplementary Table 5. Host Defense Gene Expression in Nonsmoker Club Cell Subclusters[1]

Category	Gene symbol	Gene name	Mean expression (UMI)[2]		
			CC1	CC2	CC3
Anti-inflammation	SCGB1A1	secretoglobin, family 1A, member 1	117.5	111.4	905.5
Cytokines	CXCL17	chemokine (C-X-C motif) ligand 17	6.4	7.6	9.3
	CXCL1	chemokine (C-X-C motif) ligand 1	7.6	7.5	14.1
	CXCL6	chemokine (C-X-C motif) ligand 6	2.4	2.3	7.6
Immune function	C3	complement component 3	1.2	2.4	9.9
	CD55	complement decay accelerating factor	1.5	2.4	6.8
	B2M	beta-2-microglobulin	15.9	34.9	19.4
	PIGR	polymeric immunoglobulin receptor	1.8	4.4	22.9
	TLR5	toll-like receptor 5	0.8	1.3	0.6
Anti-bacterial	LCN2	lipocalin 2	3.5	6.2	23.8
	LYZ	Lysozyme	0.2	0.2	1.3
	BPIFB1	BPI fold containing family B, member 1	5.0	3.2	58.1
Xenobiotic metabolism	GSTP1	glutathione S-transferase pi 1	14	15.2	11.6
	RDH10	retinol dehydrogenase 10 (all trans)	0.4	0.3	1.6
	AKR1C1	aldo-keto reductase family 1, member C1	0.9	0	3.7
	DHRS9	dehydrogenase/reductase member 9	0.4	0	0.9
	CYP2F1	cytochrome P450, family 2, subfamily F, polypeptide 1	0.8	0.8	3.7
	MGST1	microsomal glutathione S-transferase 1	5.6	6.2	4.8
	CYP4B1	cytochrome P450, family 4, subfamily B, polypeptide 1	4.2	2.1	4.5
Anti-protease	SERPINA1	serpin peptidase inhibitor, clade A (alpha-1 antiprotease), member 1	0	0	0.1
	SLPI	secretory leukocyte peptidase inhibitor	68.1	48.4	221.3
	WFDC2	WAP four-disulfide core domain 2	11.1	14.1	42.1
	SERPINB3	serpin peptidase inhibitor, clade B (ovalbumin), member 3	34.3	10.6	31.3
	CST3	cystatin C	3.1	2.5	4.8
Barrier function	CTNNB1	catenin, beta 1, 88kDa	1.2	1.6	1.5
	CXADR	coxsackie virus and adenovirus receptor	0.9	1.5	1.2
	CLDN1	claudin 1	3.9	5.2	1.9
	CLDN4	claudin 4	2.0	2.7	3.4
	OCLN	occludin	0.1	0	0.2
	TFF3	trefoil factor 3	3.1	0.7	11.6
	MUC1	mucin 1, cell surface associated	0.9	0.7	6.7
	MUC5B	mucin 5B, oligomeric mucus/gel-forming	0.6	0.1	8.7
	ITGAV	integrin, alpha V	0.3	1.3	0.3
	ITGB1	integrin, beta 1	1.8	3.5	1.8

[1] Genes with defense related function expressed in club cells as identified in Zuo et al (2018)[3].

[2.] Mean expression for each of the club cell subclusters; UMI = unique molecular identifiers, CC = club cell.

Supplementary Table 6. Differentially Expressed Genes of Club Cell 1 *vs* Club Cell 3: GO Term Analysis

Club Cell 1 Enriched GO Terms		
**GO term**	**Genes**	**pBH**
SRP-dependent cotranslational protein targeting to membrane	78 genes	2.3×10^{-123}
Nuclear-transcribed mRNA catabolic process, nonsense-mediated decay	80 genes	4.1×10^{-114}
rRNA processing	78 genes	1.4×10^{-83}
Cytoplasmic translation	RPL35A, RPL36A, RPL15, RPL26, RPLP0P6, RPLP2, RPL36, RPL29, RPL7, RPL22, RPL31, RPL6, RPL9, RPLP0, RPL8, RPLP1	3.4×10^{-19}
Cell-cell adhesion	ALDOA, HSP90AB1, FLRT3, RPL14, TRIM29, RPL15, S100A11, RPL24, RPL23A, EEF2, SFN, RPS2, PRDX1, RPL29, PKM, RSL1D1, RACK1, PFN1, RPS26, RPL6, RPL34, EIF3E, RPL7A, PERP, SEPT7	1.5×10^{-09}
Ribosomal small subunit biogenesis	RPS28, RPS19, RPS16, RPS17, RPS15, RPS6, RPS7, RPS24	3.1×10^{-07}
ATP biosynthetic process	ALDOA, PKM, CHCHD10, ATP5B, ATP5F1, ATP5G2, ATP5G3, ATP5H, ATP5J	1.4×10^{-06}
Hydrogen ion transmembrane transport	NDUFA4, UQCRH, COX7B, COX8A, COX7C, COX4I1, COX6C, UQCRHL, UQCRB	4.8×10^{-04}
Mitochondrial electron transport, cytochrome c to oxygen	NDUFA4, COX7B, COX8A, COX7C, COX4I1, COX6C	1.0×10^{-03}
Mitochondrial ATP synthesis coupled proton transport	ATP5B, ATP5F1, ATP5G2, ATP5G3, ATP5H, ATP5J	1.2×10^{-03}
Generation of precursor metabolites and energy	GNPDA1, ATP5B, COX8A, COX7C, COX4I1, ATPIF1, CROT, COX6C	1.4×10^{-03}

Club Cell 3 Enriched GO Terms		
**GO term**	**Genes**	**pBH**
Response to lipopolysaccharide	CXCL1, ALPL, IRAK3, FOS, PTGES, CXCL3, CXCL2, SLPI, CXCL6, SCGB1A1	0.007
Positive regulation of neutrophil chemotaxis	CXCL1, CXCL3, CXCL2, CXCL8, CD74	0.012
Response to molecule of bacterial origin	CXCL2, CXCL8, CD24, TNFAIP3	0.013
Immune response	CXCL1, HLA-DRB1, C3, CXCL3, CXCL2, CXCL8, CXCL6, CD74, TNFSF10, XBP1, SLPI, CTSC, HLA-DRA	0.034
Inflammatory response	CXCL1, NFKBIZ, FOS, ELF3, C3, CXCL3, CXCL2, S100A9, LYZ, CXCL8, CXCL6, TNFAIP3	0.045

[1] Genes with defense related function expressed in club cells as described by Zuo et al (2018)[3].

[2] pBH = Benjamini-Hochberg corrected p values.

Supplementary References

1. Zuo WL, Rostami MR, Shenoy SA, M. L, J. S, Strulovici-Barel Y, O'Beirne SL, R.J. K, Leopold PL, Mezey JG, Schymeinsky J, Quast K, Visvanathan S, Fine JS, Thomas M, Crystal RG. Cell-specific Expression of Lung Disease Risk-related Genes in the Human Small Airway Epithelium Respir Res. 2020; (in press).
2. Jones PW, Quirk FH, Baveystock CM, Littlejohns P. A self-complete measure of health status for chronic airflow limitation. The St. George's Respiratory Questionnaire. Am Rev Respir Dis. 1992; 145:1321-1327.
3. Zuo WL, Shenoy SA, Li S, O'Beirne SL, Strulovici-Barel Y, Leopold PL, Wang G, Staudt MR, Walters MS, Mason C, Kaner RJ, Mezey JG, Crystal RG. Ontogeny and Biology of Human Small Airway Epithelial Club Cells. Am J Respir Crit Care Med. 2018; 198:1375-1388.

Supplementary Figure Legends

Supplementary Figure 1. Distribution of club cell subcluster cells 1, 2, and 3 (CC1, 2 and 3, respectively) superimposed on the original t-SNE plot identifying airway cell types. Subclustering of club cells is based on a second, unsupervised clustering process and, therefore, does not necessarily correlate with features of the original clustering process that identified the group of club cells. As a result, cells corresponding to club cell subclusters 1, 2, and 3 are found scattered through the original club cell cluster where they are plotted according to the criteria that were initially used to identify club cells. PNEC = pulmonary neuroendocrine cells; APC = antigen presenting cells; NCL^{high} represents a distinct cluster of cells identified in Zuo et al[1].

Supplementary Figure 2. Reproduction of panel C from Figure 1 showing the names of the genes on the heatmap demonstrating differential gene expression among the three club cell subclusters.

Supplementary Figure 3. Comparison of expression of KRT5 and KRT7 in the secretory lineage utilizing a single club cell cluster or three club cell subclusters. Mean gene expression (UMI per cell) was plotted for cells in each cluster of the secretory lineage using either a single cluster for club cells (A, C) or the three club cell subclusters (B, D). The plots show how expression of a signature gene from the basal cell (BC) lineage, e.g., KRT5, can be expressed at high levels in a subcluster from another cell type [e.g., KRT5 expression in club cell 1 (CC1)]) even though the overall expression of KRT5 in club cells is lower than BC. Expression of KRT7, a signature gene for club cells[1] is included to document the identity of CC1 as club cells. **A.** KRT5 expression in the secretory lineage with a single club cell cluster. **B.** KRT5 expression in the secretory lineage with a three club cell clusters. **C.** KRT7 expression in the secretory lineage with a single club cell cluster. **D.** KRT7 expression in the secretory lineage with three club cell clusters.

Supplementary Figure 4. Effector club cell gene expression profile comparing smokers (red) and nonsmokers (blue) using GAGE analysis. Fold-enrichment of GO terms was plotted based on the log of the Benjamini–Hochberg corrected p value. Vertical dotted lines represent a corrected p value less than 0.05. Only enrichments that are significant in at least one of the phenotypes are shown.

Supplemental Figure 1

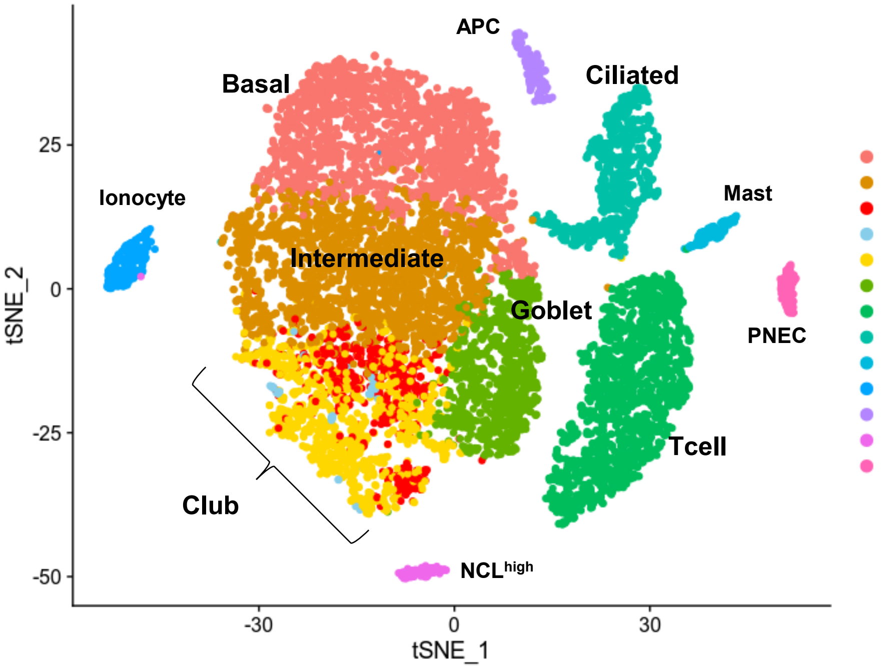

Supplemental Figure 2

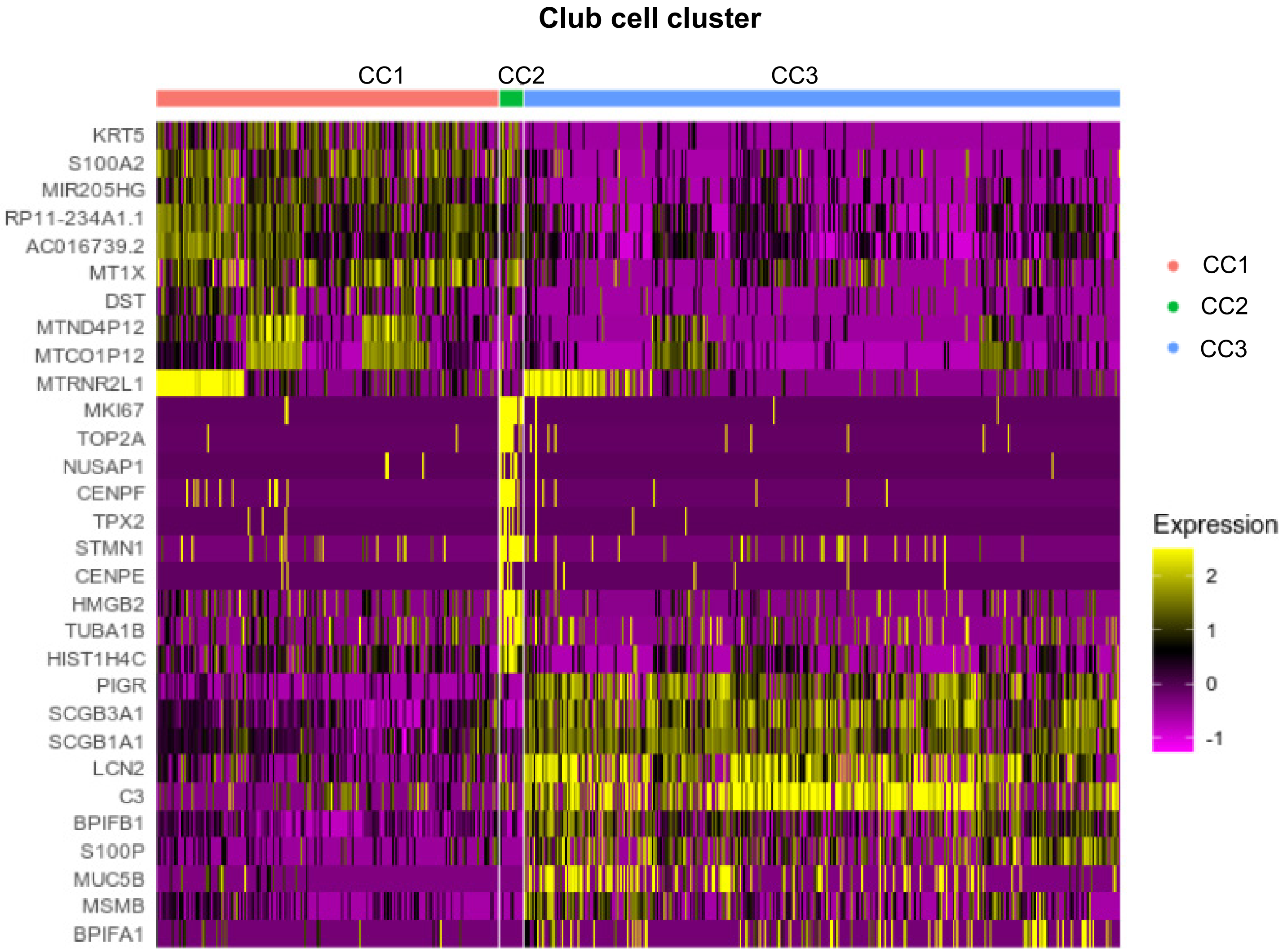

Supplemental Figure 3

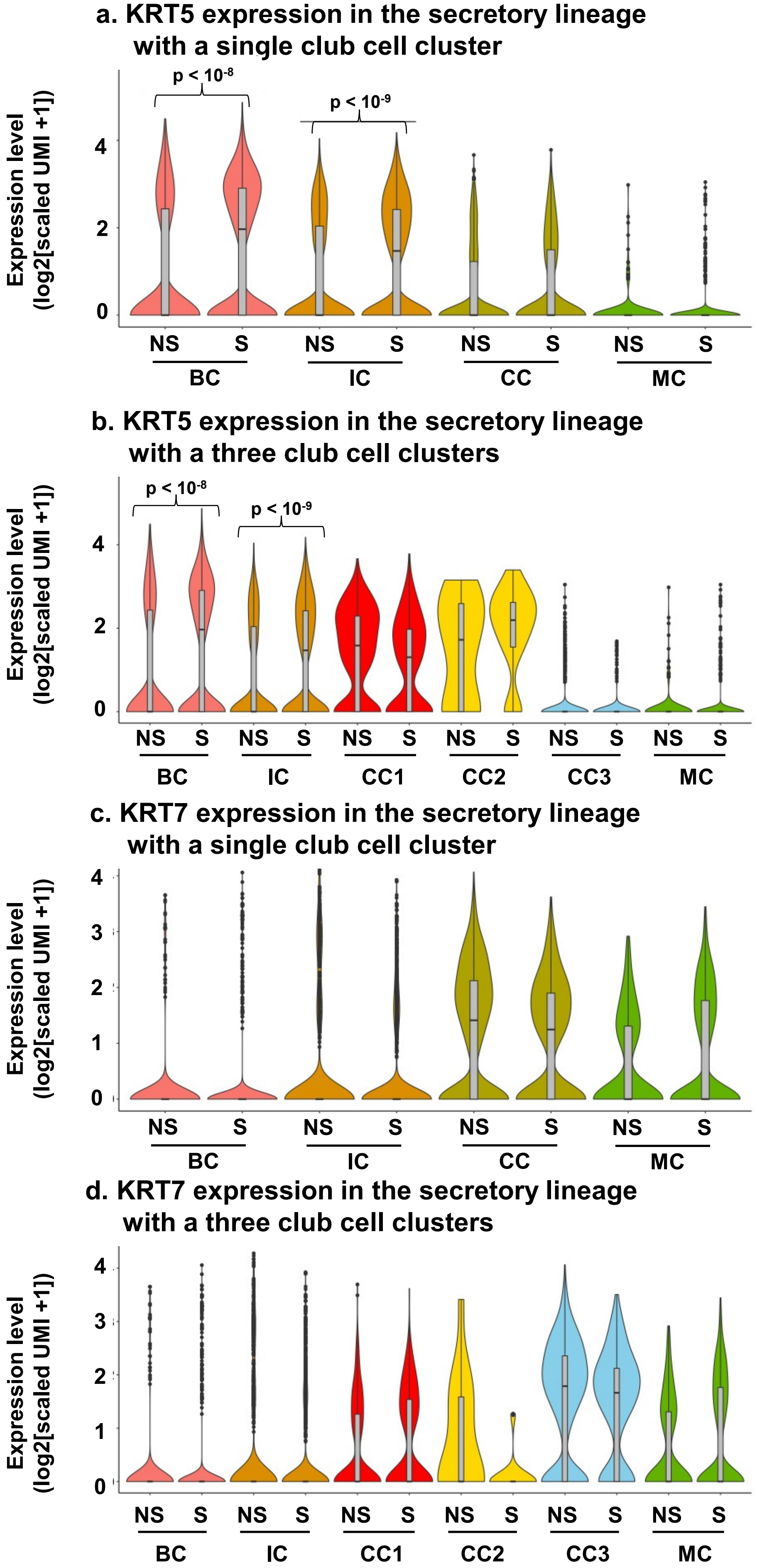

Supplemental Figure 4

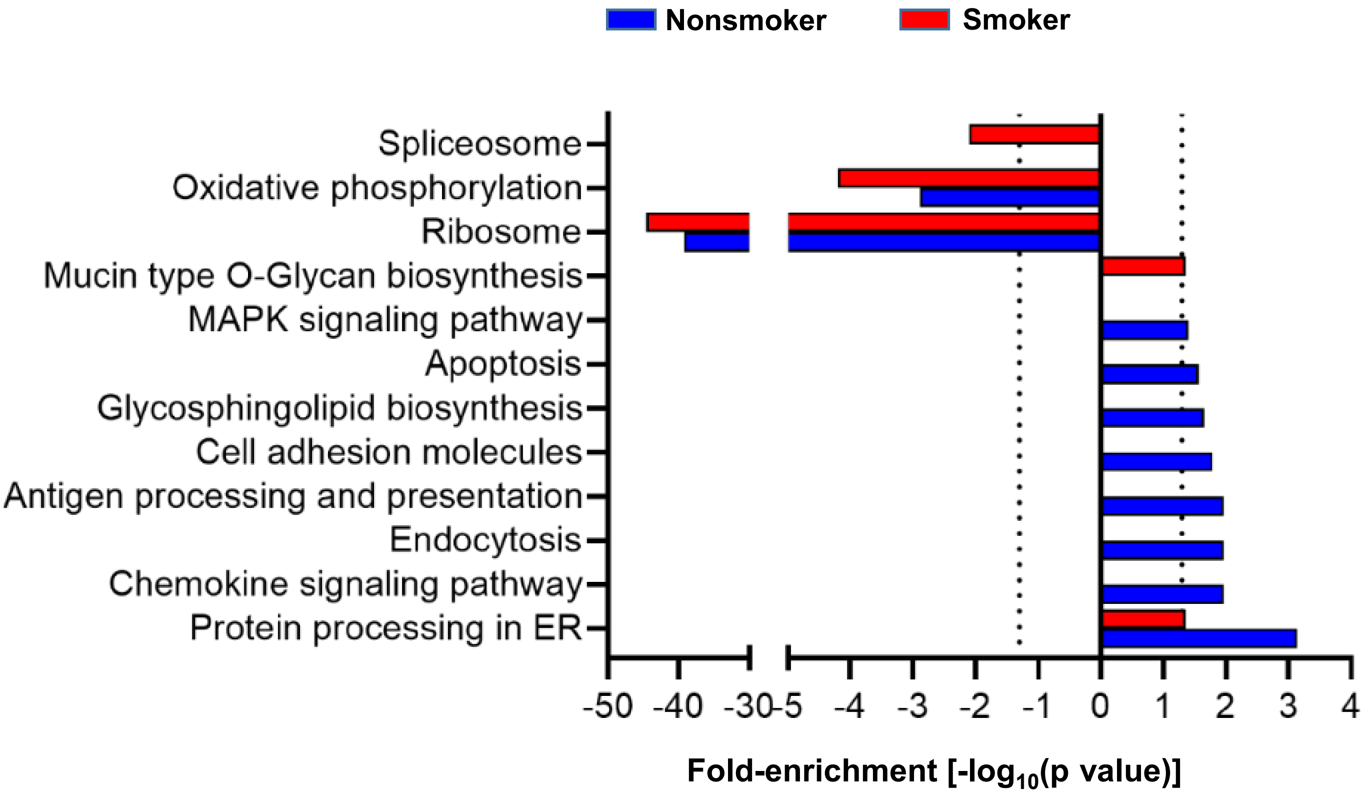

Supplement: Supplementary file 2 — Supplementary Information [file 41525_2021_237_MOESM2_ESM.pdf]
